# Long-chain acylcarnitine deficiency promotes hepatocarcinogenesis

**DOI:** 10.1016/j.apsb.2025.01.017

**Published:** 2025-01-28

**Authors:** Kaifeng Wang, Zhixian Lan, Heqi Zhou, Rong Fan, Huiyi Chen, Hongyan Liang, Qiuhong You, Xieer Liang, Ge Zeng, Rui Deng, Yu Lan, Sheng Shen, Peng Chen, Jinlin Hou, Pengcheng Bu, Jian Sun

**Affiliations:** a State Key Laboratory of Organ Failure Research; Key Laboratory of Infectious Diseases Research in South China, Ministry of Education; Guangdong Provincial Clinical Research Center for Viral Hepatitis; Guangdong Provincial Key Laboratory of Viral Hepatitis Research; Department of Infectious Diseases, Nanfang Hospital, Southern Medical University, Guangzhou 510515, China; b Department of Pathophysiology, Guangdong Provincial Key Laboratory of Proteomics, School of Basic Medical Sciences, Southern Medical University, Guangzhou 510515, China; c Key Laboratory of Epigenetic Regulation and Intervention, Institute of Biophysics, Chinese Academy of Sciences, Beijing 100101, China; d Key Laboratory of RNA Biology, Institute of Biophysics, Chinese Academy of Sciences, Beijing 100101, China; e College of Life Sciences, University of Chinese Academy of Sciences, Beijing 100049, China

**Keywords:** Hepatocellular carcinoma, Metabolomics, H3K14, CUT&Tag, Chemoprevention, Long-chain acylcarnitine, KLF6, Acetyl coenzyme A

## Abstract

Despite therapy with potent antiviral agents, chronic hepatitis B (CHB) patients remain at high risk of hepatocellular carcinoma (HCC). While metabolites have been rediscovered as active drivers of biological processes including carcinogenesis, the specific metabolites modulating HCC risk in CHB patients are largely unknown. Here, we demonstrate that baseline plasma from CHB patients who later developed HCC during follow-up exhibits growth-promoting properties in a case–control design nested within a large-scale, prospective cohort. Metabolomics analysis reveals a reduction in long-chain acylcarnitines (LCACs) in the baseline plasma of patients with HCC development. LCACs preferentially inhibit the proliferation of HCC cells *in vitro* at a physiological concentration and prevent the occurrence of HCC *in vivo* without hepatorenal toxicity. Uptake and metabolism of circulating LCACs increase the intracellular level of acetyl coenzyme A, which upregulates histone H3 Lys14 acetylation at the promoter region of *KLF6* gene and thereby activates KLF6/p21 pathway. Indeed, blocking LCAC metabolism attenuates the difference in *KLF6/p21* expression induced by baseline plasma of HCC/non-HCC patients. The deficiency of circulating LCACs represents a driver of HCC in CHB patients with viral control. These insights provide a promising direction for developing therapeutic strategies to reduce HCC risk further in the antiviral era.

## Graphical abstract

Circulating long-chain acylcarnitines deficiency promotes hepatocellular carcinoma in chronic hepatitis B patients with viral control *via* modulating histone H3 acetylation.

**Figure:**
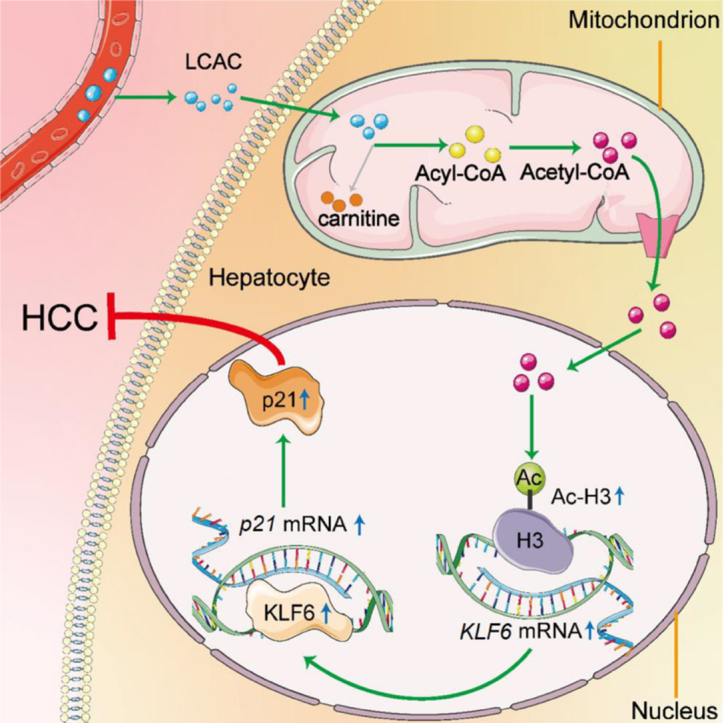

## 1 Introduction

Hepatocellular carcinoma (HCC), the predominant primary malignancy of the liver, is one of the most malignant human cancers^1^^,^^2^. Chronic infection with hepatitis B virus (HBV) plays a pivotal role in the etiology of HCC, and the prevalence of HCC is high in epidemic areas of chronic hepatitis B (CHB)^3^. Fortunately, therapy with nucleos(t)ide analogues (NAs), has proven effective in inhibiting the replication of HBV and reducing HCC risk^4^^,^^5^. However, despite the use of current first-line NAs, long-term follow-up studies have indicated that approximately 0.9%–5.4% of CHB patients with cirrhosis still develop HCC annually^6^. Therefore, beyond antiviral treatment, identifying new targets for the prevention of HCC remains an unmet medical need.

In recent years, metabolites have been rediscovered as signaling molecules^7^^,^^8^. Cells can perceive changes in metabolites in the external environment and perform correspondingly to response *via* reorganizing the metabolic network and modulating cell signaling^7^, ^8^, ^9^. Particularly, a series of studies have provided compelling evidence that metabolite signaling affects the biological behavior of tumors^10^^,^^11^. For example, Gomes et al.^12^ have revealed that accumulation of methylmalonic acid makes the blood of older people favor cancer aggressiveness *via* inducing SOX4 expression. Indeed, identifying the bioactive metabolites involved in HCC development might provide new clues for HCC prevention. Several prospective studies have recently established the statistical association between circulating metabolites and HCC risk^13^, ^14^, ^15^. However, it is still unclear whether specific metabolites have modulated the HCC risk of patients with chronic liver disease.

Long-chain acylcarnitines (LCACs) are esters of l-carnitine and long-chain fatty acids and function as transporters of long-chain acyl groups from the cytosol into the mitochondrial matrix for *β*-oxidation. Although several studies have evaluated the association between blood levels of LCAC and HCC risk, the results are conflicting^16^, ^17^, ^18^. Recently, Cheng et al.^19^ reported that accumulated LCACs in HCC tissues might induce dysregulation of invariant natural killer cells, while the comprehensive effect of LCACs on hepatocarcinogenesis was not evaluated. Hence, the role of circulating LCACs in hepatocarcinogenesis warrants further investigation.

In this study, utilizing a large-scale, prospective CHB cohort, we demonstrated that baseline plasma from those who developed HCC during follow-up was growth promotive. Through unbiased metabolomics analysis, we revealed that LCACs were reduced in baseline plasma from patients with HCC development. Further functional studies showed that LCACs exerted anti-tumor activity in HCC by increasing histone H3 acetylation in the KLF6 promoter region. Together, our findings reveal that in addition to antiviral therapy, a healthy metabolome and specifically, LCACs, is a promising target to further prevent HCC in CHB patients.

## 2 Materials and methods

### 2.1 Human samples

This was a nested case–control study in a real-life, prospective cohort (Clinical Trial Number 02167503). Between May 2014 and January 2018, 3368 CHB patients were recruited from Nanfang Hospital, Southern Medical University. All patients received NAs treatment and achieved complete viral suppression (HBV DNA <20 IU/mL). Patients were excluded in cases of coinfection with hepatitis C, hepatitis D or human immunodeficiency virus, interferon-*α* treatment, or diagnosis with HCC before study enrollment. We additionally excluded patients who received interferon-*α* treatment during follow-up. Every six months, patients were assessed for the development of HCC with liver ultrasound. New cases of HCC identified based on liver ultrasound were confirmed *via* histopathology or imaging (CT or MRI). During a median follow-up of 43.5 months, a total of 98 CHB patients developed HCC. After excluding 21 HCC cases diagnosed within the first six months of follow-up and 1 case with unavailable baseline plasma, 76 HCC cases were included. For each case, a control matched for age, sex, total bilirubin, platelet, albumin, and body mass index was selected from the remaining CHB patients who did not develop HCC. The baseline plasma of cases and controls were used for metabolomics analyses.

The study protocol was approved by the ethics committee of Nanfang Hospital, Southern Medical University (Ethical Committee Approval Code: NEFC-2014-017). All patients signed informed consent before enrollment.

### 2.2 Metabolite detection

For untargeted metabolomics, the human plasma samples were sent to Shanghai Applied Protein Technology (Shanghai, China) for analysis. Human plasma samples stored at −80 °C were pretreated according to previously reported procedures^20^. All plasma samples were thawed at 4 °C overnight and vortexed for 30 s. Next, a 100 μL sample aliquot was mixed with 200 μL acetonitrile and 200 μL methanol for deproteinization. The mixture was incubated at −20 °C for 1 h, followed by vortexing (30 s) and centrifugation (14,000×*g*, 4 °C for 20 min). Supernatant fractions were collected, dried under a stream of nitrogen at 45 °C, and redissolved in 50% acetonitrile–water solution for mass spectrometry analysis. Separation and analysis were performed using an Agilent 1290 Infinity Liquid chromatography column (Agilent Technologies, Santa Clara, USA) and an AB Triple TOF 5600 mass spectrometer (AB SCIEX, Framingham, USA), respectively.

Quantitative measurement of LCACs was performed using a Q300 kit at Human Metabolomics Institute, Inc. (Shenzhen, China) based on a previously published method^21^. Plasma samples were slowly dissolved at 4 °C, and 25 μL aliquots were mixed with 120 μL pre-cooled methanol solution containing internal standard. The mixture was stirred for 5 min and centrifuged at a low temperature for 4000×*g* and 30 min. Cell samples were slowly dissolved on ice, mixed with 150 μL pre-cooled methanol solution containing internal standard, ultrasonicated, and centrifugated at 18,000×*g* for 30 min at 4 °C. The supernatant (30 μL) was further mixed with 20 μL derivatization reagent at 30 °C for 60 min. A 330 μL aliquot of 50% pre-cooled methanol solution was used to resuspend the derivative mixture, followed by incubation at −20 °C for 20 min and centrifugation at 4 °C at 4000×*g* for 30 min. A 135 μL aliquot of supernatant was mixed with a 10 μL internal standard. Ultra-performance liquid chromatography coupled to tandem mass spectrometry (UPLC–MS/MS) system (ACQUITY UPLC-Xevo TQ-S, Waters Corp., Milford, USA) was used to quantitate the metabolite. Formic acid (0.1%) and acetonitrile and isopropanol solution (7:3) were used as mobile phase A and mobile phase B, respectively.

The levels of acetyl-CoA and CoA were analyzed using liquid chromatography-tandem MS analysis for targeted analysis. Cells were inoculated on a 10 cm^2^ dish. At ∼70% confluence, cells were treated with LCAC-16:0 for 24 h. Cells were washed with ice-cold PBS that was aspirated off the dish, and cells were washed again with ice-cold physiological saline that was aspirated off. Ice-cold physiological saline (1 mL) was added to the culture dish, and cells were scraped into the cold physiological saline, snap-frozen in liquid nitrogen, and transferred to −80 °C until analysis. Samples were thawed on ice and centrifuged at 4 °C, cell pellets were resuspended with acetonitrile/methanol/water mixture (2:2:1, 1 mL) and ultrasonicated at 4 °C, followed by incubation at −20 °C for 20 min and centrifugation at 4 °C at 15,800×*g* for 30 min. The supernatant was transferred to clean tubes dried under a stream of nitrogen at 45 °C, redissolved in 50% acetonitrile–water solution, and ultrasonicated at 4 °C, followed by centrifugation at 4 °C at 15,800×*g* for 30 min. The supernatant was collected and subjected Q-TRAP 6500 mass spectrometer (AB SCIEX, Framingham, USA), and separation was achieved on a UPLC BEH Amide column (2.1 mm × 5 mm, Waters Corp., Milford, USA). Ammonium acetate ammonium hydroxide (1:1, 20 mmol/L) and acetonitrile were used as mobile phase A and mobile phase B, respectively.

### 2.3 HCC models, treatment, and analysis

For the xenograft mouse model, SMMC-7721 or MHCC97H cells (5 × 10^6^)/100 μL PBS were subcutaneously inoculated into the right flanks of BALB/c nude mice randomized into two groups. After one week, the experimental groups received an intraperitoneal injection of LCAC-16:0 (25 mg/kg/day) every day while the control group received PBS daily for 14 days. Tumor-bearing mice were sacrificed on Day 21 after inoculation. Tumor volumes (*V*) were measured using a digital caliper and calculated according to Eq. (1):

(1) *V* = *L* × *W*^2^/2

where *L* is length, *W* is width.

For the orthotopic mouse model, 1 × 10^6^ Hepa1-6 cells were inoculated into the liver of C57BL/6 mice. After one week, the experimental groups received an intraperitoneal injection of LCAC-16:0 (25 mg/kg/day) every day while the control group received PBS daily for 14 days. Tumor-bearing mice were sacrificed on Day 21 after inoculation. Tumor volumes were measured using a digital caliper and calculated according to Eq. (1).

For the oncogene mouse model, 6 to 8-week-old C57BL/6J mice were administered 20 μg pT3-EF1*α*-myr-AKT, 20 μg NRasV12/pT2-CAGGS, and 1.6 μg pCMV/SB by hydrodynamic injection (HDI). HDI was performed as described^22^^,^^23^. After one week, the experimental groups received an intraperitoneal injection of LCAC-16:0 (25 mg/kg/day) every day while the control group received PBS daily for 28 days. All mice were sacrificed on Day 35 after HDI, and liver and blood samples were collected for further experiments. Liver samples were further weighed and digital images were obtained to detect HCC development. Two independent researchers evaluated macroscopic malignant nodules (diameter >0.5 mm).

For the diethylnitrosamine (DEN)/tetrachloromethane (CCl_4_)-induced mouse model, C57BL/6J mice were intraperitoneally administered DEN (Sigma, Saint Louis, MO, USA; 25 mg/kg, dissolved in PBS) once on Day 14 after birth, followed by weekly injections of CCl_4_ (Macklin, Shanghai, China; 0.5 mL/kg, dissolved in olive oil) starting at 4 weeks of age. Mice received PBS or LCAC-16:0 (25 mg/kg/day) daily for 6 weeks *via* intraperitoneal injection, beginning at 12 weeks of age. Body weights were measured every week after the PBS/LCAC-16:0 intervention. All mice were sacrificed at 18 weeks and liver samples were collected for further experiments. Liver samples were further weighed and digital images were obtained to detect HCC development. Two independent researchers evaluated macroscopic malignant nodules (diameter >0.5 mm).

### 2.4 ^13^C-labeled LCAC-16:0 tracing analysis

Isotope labeling experiments were performed as described previously, with minor modifications^24^. ^13^C-1,2,3,4-LCAC-16:0 hydrochloride (662127-VAR) was purchased from Sigma. Briefly, Huh7 cells were seeded in six-well plates. After 24 h, the medium was replaced with Krebs Ringer Buffer (135 mmol/L NaCl, 5 mmol/L KCl, 1 mmol/L MgSO_4_, 4 mmol/L K_2_HPO_4_, 5.5 mmol/L Glucose, 20 mmol/L HEPES, 1 mmol/L CaCl_2_, pH 7.4) containing 7.5 μmol/L ^12^C-LCAC-16:0 or 7.5 μmol/L ^13^C-1,2,3,4- LCAC-16:0 hydrochloride. After a 6 h incubation period, cells were washed three times with pre-cold PBS and collected for metabolic tracer analysis. Afterward, the metabolites were extracted and subjected to Metabo-Profile (Shanghai, China) analysis.

### 2.5 Cleavage under targets and Tagmentation (CUT&Tag)

The CUT&Tag assay was performed using the Hyperactive In-Situ ChIP Library Prep Kit for Illumina (Vazyme Biotech Co., Nanjing, China) according to the manufacturer's instructions. Briefly, cells were sequentially incubated with ConA Beads, primary antibody (anti-H3K14ac antibody, ABclonal, Wuhan, China), secondary antibody (IgG, ABclonal, Wuhan, China), and Hyperactive pA-Tn5 Transposase. The fragmented DNA was extracted from the samples and amplified by PCR. After an assessment using the Agilent 2100 Bioanalyzer (Agilent Technologies, Santa Clara, CA, USA), these libraries were sequenced on the Illumina NovaSeq6000 platform (Illumina, San Diego, CA, USA), and 150 bp paired-end reads were generated for analysis.

### 2.6 Chromatin immunoprecipitation (ChIP)

ChIP assays were conducted with a ChIP kit (BersinBio, Guangzhou, China). Briefly, 2 × 10^7^ cells were crosslinked with 1% formaldehyde and sonicated for 15 min at 35% power to reduce the average DNA length to 200–600 bp. Samples were precleared using protein A/G beads for 1 h, followed by overnight incubation at 4 °C with anti-histone H3K14 acetylation (ABclonal, Wuhan, China). The next day, samples and magnetic beads were incubated for 30 min at room temperature. ChIP DNA products were obtained following washing, elution, de-crosslinking, and extraction steps. DNA template enrichment was analyzed *via* agarose gel electrophoresis and qPCR using primers specific for each target gene promoter. The primers used are listed in Supporting Information Table S1.

### 2.7 Statistical analysis

To evaluate the differences between groups, we employed one-way ANOVA or Wilcoxon's rank-sum test for continuous variables, and chi-square test for categorical variables. For time-to-event analysis, cumulative HCC incidences were calculated using the Kaplan–Meier method, and groups were compared with the log-rank test. Univariable (unadjusted) and multivariable (adjusted) Cox regression models were used to estimate HR for HCC at a 4.5-year follow-up and corresponding 95% CI. In a multivariable model, we adjusted traditional HCC risk factors including age, sex, total bilirubin, platelets, albumin, and elevated alanine aminotransferase (ALT). All data are presented as mean ± standard error of mean (SEM). Statistical tests used to compare conditions are indicated in figure legends. GraphPad PRISM version 10.0 and R 3.5.3 were used for the generation of graphs and statistics. Differences were considered statistically significant at *P* < 0.05 *vs.* Control, expressed as ∗*P* < 0.05 *vs.* Control; ∗∗*P* < 0.01 *vs.* Control; ∗∗∗*P* < 0.001 *vs.* Control; ∗∗∗∗*P* < 0.0001 *vs.* Control.

Additional methods are provided in the Supporting Information.

## 3 Results

### 3.1 Baseline plasma from patients who developed HCC is growth-promotive for liver cancer cells

Among 3368 CHB patients who achieved complete viral suppression (HBV DNA <20 IU/mL), 98 developed HCC during a median follow-up of 43.5 months. After excluding 21 HCC cases diagnosed in the first six months and one case without available baseline plasma, we enrolled a total of 76 patients with HCC and 76 controls without HCC using propensity score matching (Supporting Information Fig. S1A). Baseline characteristics were comparable between the two groups, except for ALT levels (Supporting Information Table S2).

**Figure 1:**
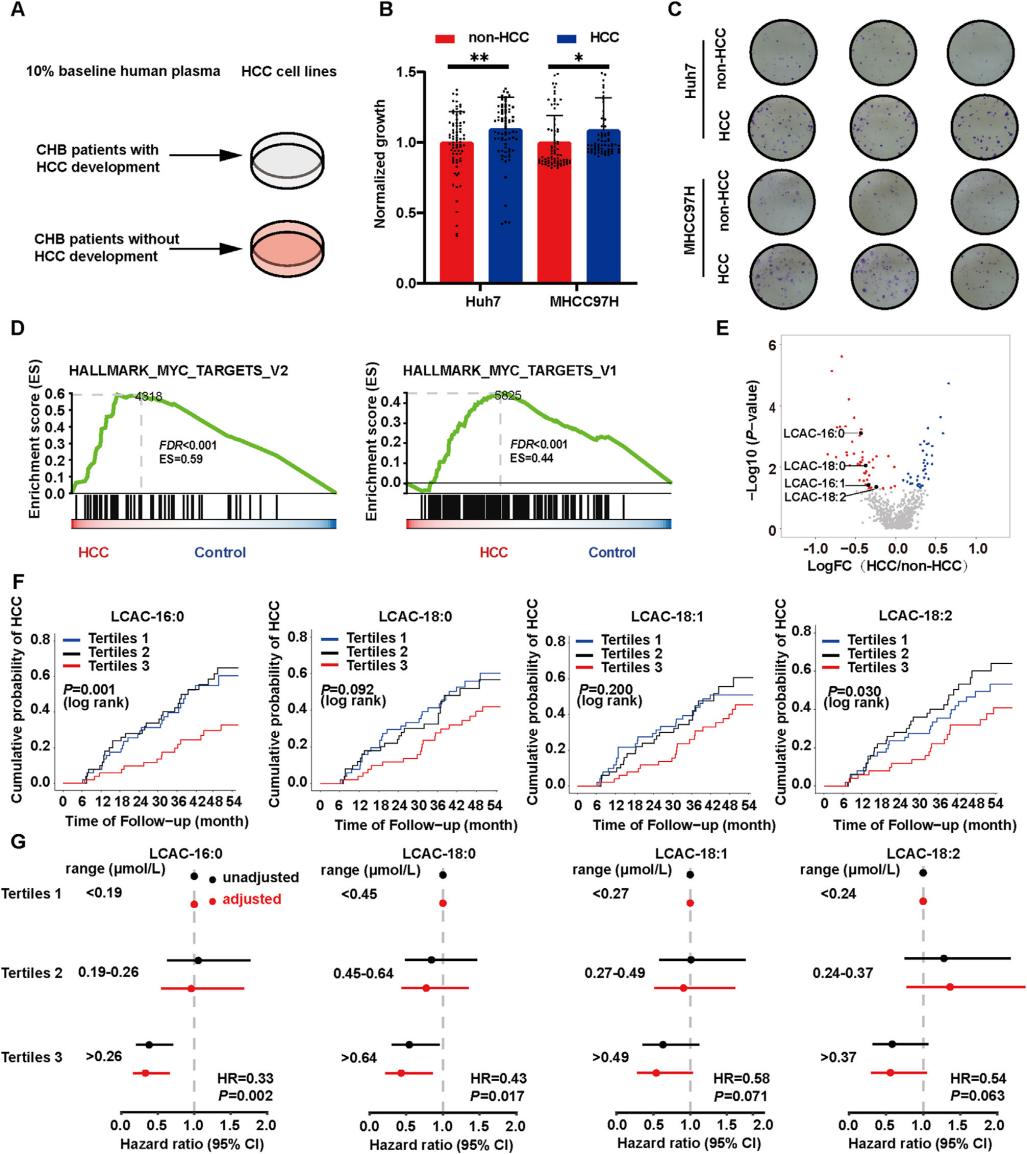
Long-chain acylcarnitines (LCACs) are negatively correlated with hepatocellular carcinoma (HCC) risk. (A) Diagram showing experimental design (see Methods). (B) Relative cell viability of cells cultured using 10% baseline plasma of chronic hepatitis B (CHB) patients (non-HCC, *n* = 76; HCC, *n* = 76). (C) Colony-forming assays of cells incubated with 10% baseline plasma of CHB patients (*n* = 3). (D) The top 2 signaling pathways induced by baseline plasma from CHB patients who developed HCC in Huh7 cells. (E) Volcano plot of LC–MS-based metabolomics from the plasma of CHB patients (non-HCC, *n* = 76; HCC, *n* = 76). (F) Kaplan–Meier estimates and the risk of HCC ranked according to LCAC tertile levels (non-HCC, *n* = 76; HCC, *n* = 76). (G) Risk of HCC by 4.5 years according to LCAC tertile levels using a multivariable Cox proportional hazard model. (non-HCC, *n* = 76; HCC, *n* = 76). Unadjusted hazard ratio (black) and adjusted model (age, sex, total bilirubin, platelets, albumin, and elevated ALT; red). The line length indicates the 95% confidence interval. Data are presented as mean ± SEM. ∗*P* < 0.05, ∗∗*P* < 0.01. *P* values were calculated based on one-way ANOVA (B), log-rank test (F), or Wald test (G).

To explore the potential roles of circulating metabolites in the development of HCC, we used baseline plasma from the two groups of patients to culture HCC cells Huh7 and MHCC97H. Intriguingly, we observed that baseline plasma from patients who later developed HCC was favorable to HCC cell proliferation and clone formation ability (Fig. 1A–C). We then performed RNA-seq on Huh7 cells incubated with baseline plasma from both groups. Gene set enrichment analysis (GSEA) revealed that baseline plasma from the HCC group induced genes involved in cell cycle progression (Fig. 1D). These findings suggest that baseline plasma from patients who developed HCC during follow-up possesses growth promotive properties.

### 3.2 Baseline plasma-derived LCACs are negatively associated with HCC risk

To elucidate the inherent differences in baseline plasma from the two groups, we conducted a non-targeted metabolomics analysis to examine plasma metabolite compositions. Among the 637 annotated compounds, 53 were significantly upregulated, and 39 were downregulated in the baseline plasma of HCC patients (*P* < 0.05). Notably, several LCACs showed a significant reduction in the HCC group (Fig. 1E and Fig. S1B–S1G).

While non-targeted metabolomics analyses provide valuable insights, they are inherently semiquantitative^25^. To validate and quantify our observations, we conducted targeted mass spectrometry analyses for palmitoylcarnitine (LCAC-16:0), stearoylcarnitine (LCAC-18:0), oleoylcarnitine (LCAC-18:1) and linoleylcarnitine (LCAC-18:2) (Fig. S1H). Plasma LCAC levels were positively correlated with liver cirrhosis and total bilirubin, and negatively correlated with albumin (Fig. S1I). Kaplan–Meier survival analyses showed negative correlations between baseline LCAC levels and HCC risk (Fig. 1F). Specifically, individuals with LCAC-16:0 (hazard ratio [HR], 0.33; 95% confidence interval [CI], 0.16–0.66), LCAC-18:0 (HR, 0.43; 95% CI, 0.21–0.86), LCAC-18:1 (HR, 0.58; 95% CI, 0.30–1.08) or LCAC-18:2 (HR, 0.54; 95% CI, 0.28–1.04) levels in the third tertiles exhibited a decreased risk of HCC (Fig. 1G). Moreover, higher LCAC levels remained an independent predictor of incident HCC risk even after adjusting for traditional HCC risk factors (Fig. 1G).

### 3.3 LCACs inhibit liver tumorigenesis

**Figure 2:**
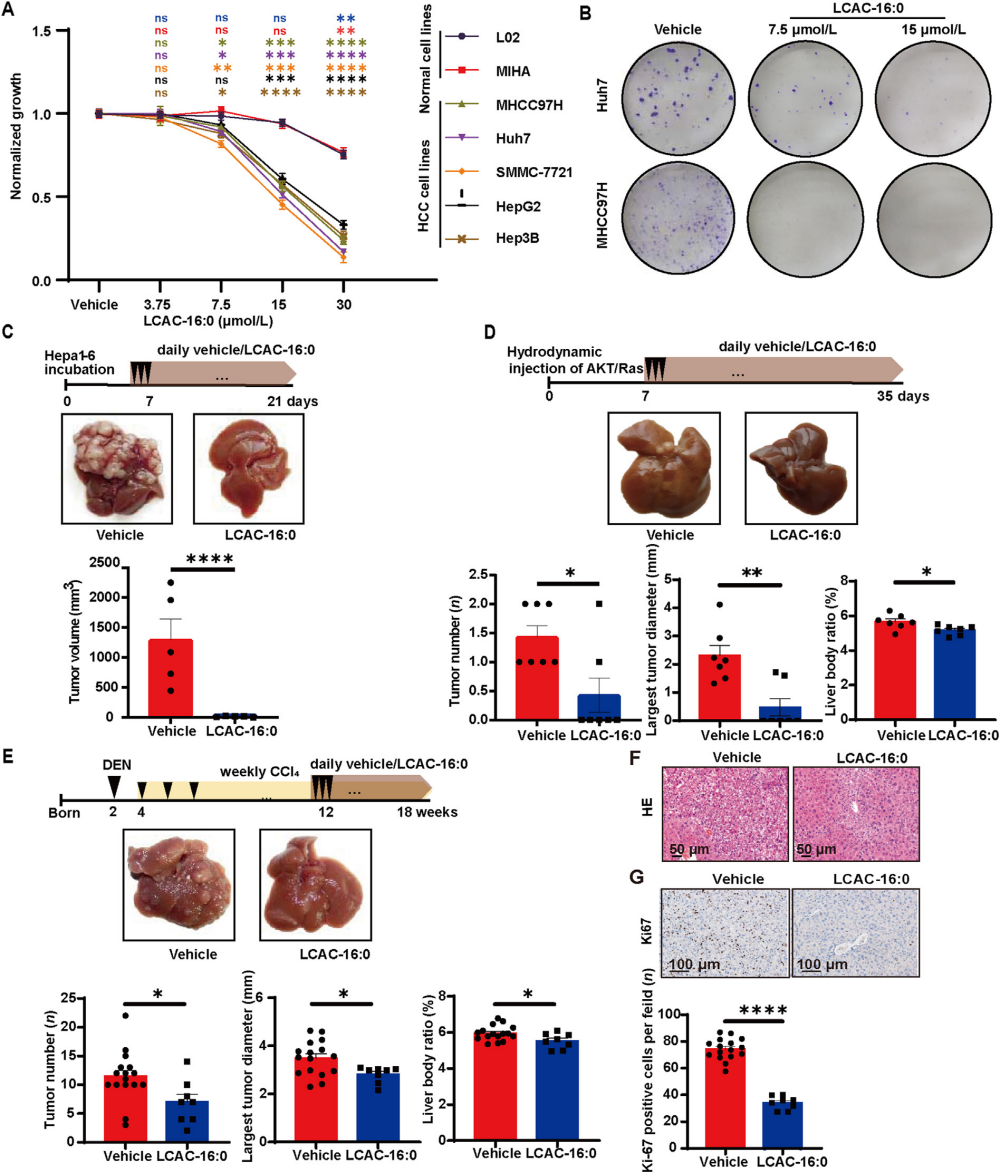
LCACs inhibit hepatocarcinogenesis. (A) Dose curve of a panel of cell lines treated LCAC-16:0 for 120 h (*n* = 3). All tested doses were compared to the vehicle group. (B) Colony-forming assays of cells treated with the indicated concentration of LCAC-16:0. (C) Mice bearing Hepa1-6 hepatoma were treated with LCAC-16:0. Representative images are shown (*n* = 5 for Vehicle, *n* = 5 for LCAC-16:0). (D) AKT/Ras mice were treated with LCAC-16:0. Representative images are shown (*n* = 7 for Vehicle, *n* = 7 for LCAC-16:0). (E) C57BL/6 mice were treated with diethylnitrosamine (DEN) plus tetrachloromethane (CCl_4_) to induce spontaneous hepatoma. Thereafter, the mice were treated with LCAC-16:0. Representative images are shown (*n* = 16 for Vehicle, *n* = 8 for LCAC-16:0). (F) Hepatic lobule structure of the DEN/CCl_4_ mice. (G) Hepatic expression of Ki67 in DEN/CCl_4_ mice. Data are presented as mean ± SEM. ∗*P* < 0.05, ∗∗*P* < 0.01, ∗∗∗*P* < 0.001, ∗∗∗∗*P* < 0.0001 *vs*. vehicle; ns, no significance. *P* values were calculated based on one-way ANOVA.

To explore the physiological function of LCACs *in vitro*, we exposed HCC (Huh7, MHCC97H, SMMC-7721, HepG2, and HepG2) and immortalized hepatocyte cell lines (MIHA and L02) to LCACs. LCACs inhibited the proliferation of HCC cells at a dose of 3.75–7.5 μmol/L (Fig. 2A and Supporting Information Fig. S2A–S2E). What should be noted, the absolute concentration of LCAC (the sum of LCAC-16:0, LCAC-18:0, LCAC-18:1, and LCAC-18:2) in the plasma of 152 CHB patients was 0.34–5.72 μmol/L (Fig. S2F), indicating that LCACs possess anti-tumor effect at a physiological concentration. Notably, immortalized hepatocyte cell lines exhibited significantly lower sensitivity to LCACs compared to HCC cell lines (Fig. 2A and Fig. S2A–S2E). The colony formation assay showed that LCAC-16:0 significantly inhibited the clone formation ability of HCC cells (Fig. 2B). We next used “human-in-mouse” xeno-transplantation HCC models to evaluate the effect of LCACs on HCC growth *in vivo*. As expected, intraperitoneal administration of LCAC-16:0 (25 mg/kg/day) significantly inhibited tumor growth in both SMMC-7721 and MHCC97H nude mouse xenograft models (Fig. S2G–S2I). Consistently, in mice bearing an orthotopic Hepa1-6 cell-derived hepatoma, LCAC-16:0 (25 mg/kg/day) also effectively suppressed hepatoma growth (Fig. 2C).

Further, we evaluated the effect of LCACs on hepatocarcinogenesis in a primary HCC mouse model induced through hydrodynamic delivery of the Sleeping Beauty transposon system with oncogene *Ras* and *Akt*^26^. We observed that mice treated with LCAC-16:0 (25 mg/kg/day) displayed fewer and smaller tumors compared to the control mice (Fig. 2D). Besides, LCAC-16:0 resulted in a reduction of liver weight without a change in body weight, leading to decreased liver body ratio (Fig. 2D and Fig. S2J). In addition, we established another primary HCC mouse model induced by DEN in combination with CCl_4_. This model incorporates chronic injury, inflammation, fibrosis, and elevated endotoxin levels mediated by CCl_4_, sharing several characteristics with the microenvironment of human HCC^27^. The mice were administered with an intraperitoneal injection of LCAC-16:0 (25 mg/kg/day) for 6 weeks^28^. Similar to the observation in AKT/Ras mice, although the body weight was similar between groups, fewer and smaller tumors, decreased liver weight, and liver body weight ratio were found in treated mice (Fig. 2E and Fig. S2K). Consistently, the hepatic lobule structures of the control mice were more disordered than those of the LCAC-16:0 treated DEN/CCl_4_ mice (Fig. 2F), and the expression of Ki67 was decreased in the treated group (Fig. 2G). Collectively, our data suggest that LCACs are effective in preventing HCC tumorigenesis.

Examination of the liver and kidney functions of AKT/Ras mice revealed no significant differences in serum ALT, aspartate aminotransferase (AST), creatinine, and urea between LCAC-16:0-treated and control group, suggesting that LCAC-16:0 administered at a dose of 25 mg/kg/day does not induce obvious hepatorenal toxicity in mice (Fig. S2L).

### 3.4 Uptake and metabolism of LCACs are required for their anti-tumor effect

**Figure 3:**
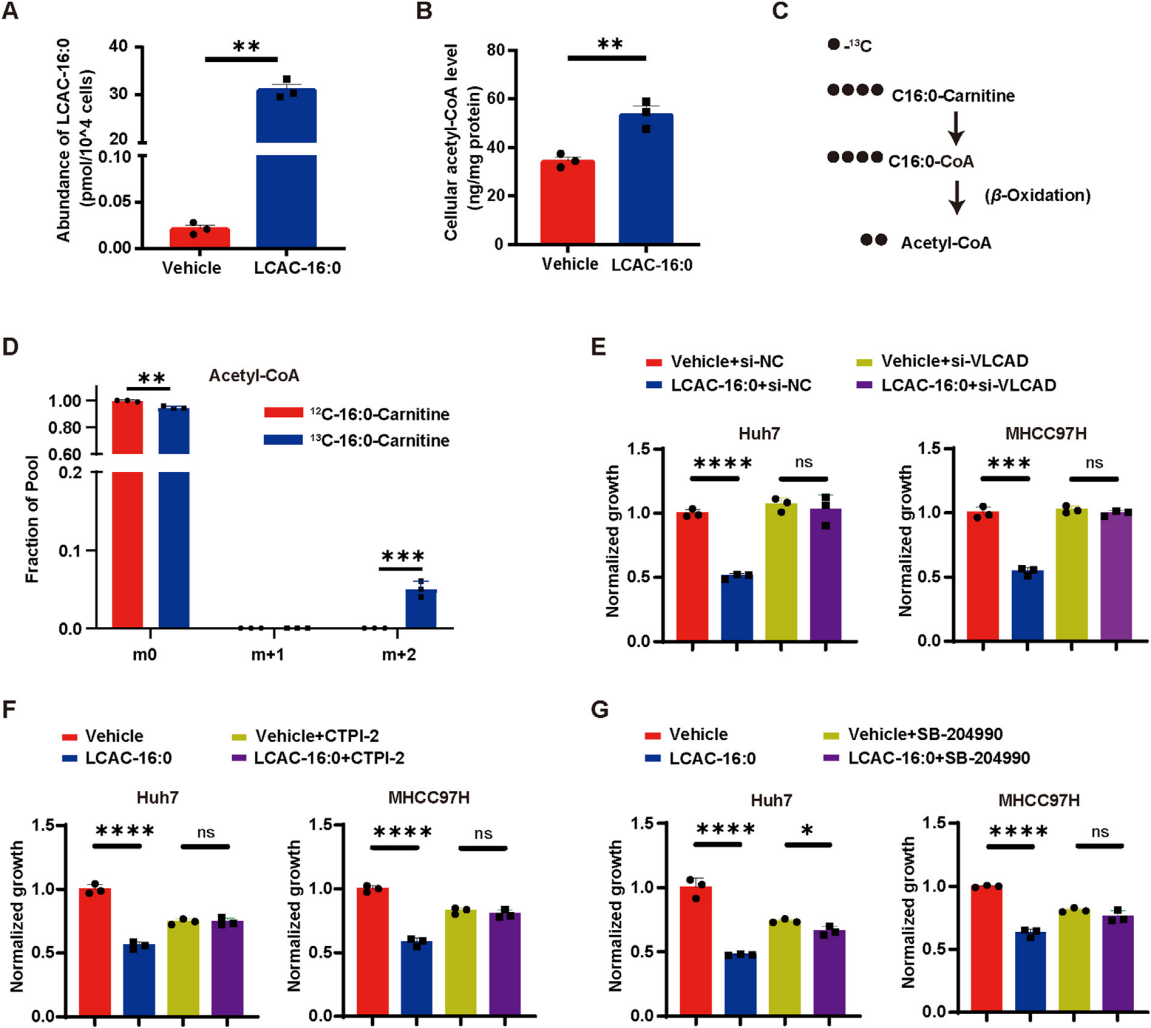
Uptake and metabolism of LCACs are required for their anti-tumor effect. (A) Intracellular LCAC-16:0 levels in Huh7 cells after incubation with 7.5 μmol/L LCAC-16:0 for 24 h (*n* = 3). (B) Intracellular acetyl-CoA levels in Huh7 cells after incubation with 7.5 μmol/L LCAC-16:0 for 24 h (*n* = 3). (C) Schematic of ^13^C-(1,2,3,4)-LCAC-16:0 facilitating incorporation of ^13^C into acetyl-CoA. (D) Isotopic tracing analysis of ^13^C-(1,2,3,4)-LCAC-16:0 in Huh7 cells (*n* = 3). (E) Inhibitory effect of LCAC-16:0 (15 μmol/L for 120 h) on HCC cells upon siRNA-mediated inhibition of very long-chain acyl-coenzyme A dehydrogenase (VLCAD) expression (*n* = 3). (F) Inhibitory effect of LCAC-16:0 (15 μmol/L for 120 h) on HCC cells upon CTPI-2 mediated inhibition of transport of acetyl-CoA out of mitochondria (*n* = 3). (G) Inhibitory effect of LCAC-16:0 (15 μmol/L for 120 h) on HCC cells upon SB-204990 mediated depletion of cytoplasmic acetyl-CoA (*n* = 3). Data are presented as mean ± SEM. ∗*P* < 0.05, ∗∗*P* < 0.01, ∗∗∗*P* < 0.001, ∗∗∗∗*P* < 0.0001; ns, no significance. *P* values were calculated based on one-way ANOVA.

LCACs, formed in the mitochondria as intermediates of *β*-oxidation, are typically metabolized into acetyl coenzyme A (acetyl-CoA)^29^. However, it remains unclear whether hepatocytes uptake extracellular LCACs. Our findings showed that incubation with LCAC-16:0 led to a remarkable increase of intracellular LCAC-16:0, acetyl-CoA, and acetyl-CoA/CoA ratio in Huh7 cells (Fig. 3A and B, Supporting Information Fig. S3A). Meanwhile, Huh7 cells were treated with medium containing ^12^C-LCAC-16:0 or ^13^C-(1,2,3,4)-LCAC-16:0, and incorporation of ^13^C into intracellular acetyl-CoA and citrate was measured through liquid metabolic flux detection. The m+2 isotopomer of acetyl-CoA and citrate was detected, indicating the LCAC-16:0 was converted to acetyl-CoA (Fig. 3C and D, Fig. S3B). Very long-chain acyl-coenzyme A dehydrogenase (VLCAD), an inner mitochondrial membrane enzyme, catalyzes the rate-limiting step of conversion of mitochondrial LCACs into acetyl-CoA^30^. We found that the knockdown of VLCAD by siRNA (Fig. S3C) abolished the increase of acetyl-CoA and the anti-proliferation effect of LCAC-16:0 (Fig. 3E and Fig. S3D). Moreover, overexpression of VLCAD reinforced the anti-proliferation effect (Fig. S3E). Additionally, we employed CTPI-2, an inhibitor of the transport of acetyl-CoA produced by *β* oxidation out of mitochondria, by blocking the mitochondrial citrate carrier SLC25A1^31^. Co-treatment with CTPI-2 abolished the inhibitory effect of LCAC-16:0 on HCC cells (Fig. 3F). Finally, the depletion of cytoplasmic acetyl-CoA *via* SB-204990, a specific inhibitor of ATP citrate lyase (ACLY) enzyme^32^^,^^33^, also abrogated the anti-proliferation effect (Fig. 3G). Collectively, our data demonstrate that uptake and metabolism of LCACs are required for their anti-tumor effect.

### 3.5 LCACs suppress HCC by modulating H3 histone acetylation

**Figure 4:**
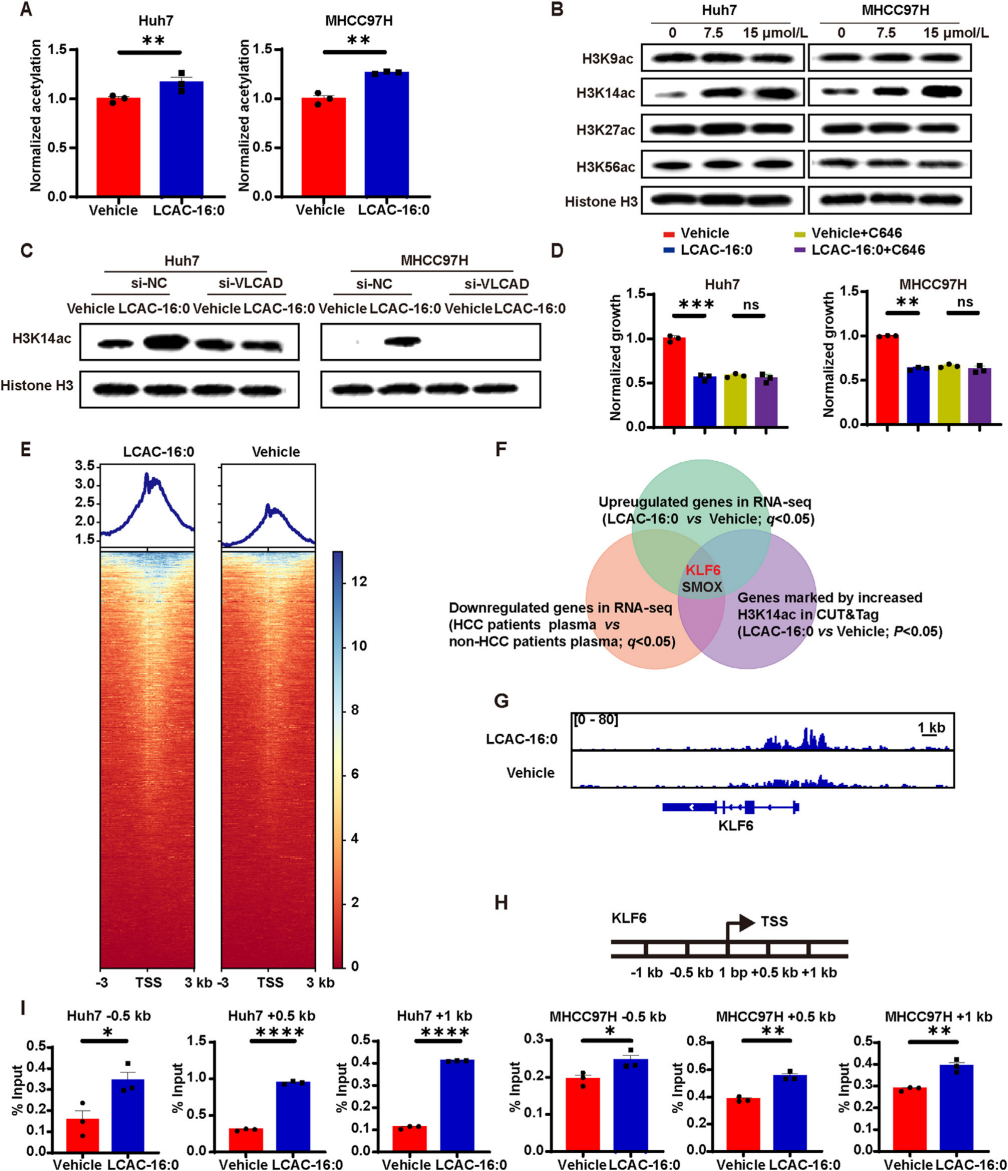
LCACs act through modulating H3 histone acetylation. (A) ELISA showing acetylation levels of H3 in HCC cells treated with 7.5 μmol/L LCAC-16:0 for 24 h. (B) Western blot showing acetylation levels of H3K14, H3K27, and H3K56 of HCC cells treated with the indicated concentration of LCAC-16:0 for 24 h. (C) Western blot showing H3K14ac levels in HCC cells with si-NC or si-VLCAD in the presence or absence of LCAC-16:0 (15 μmol/L for 24 h) treatment. (D) Inhibitory effect of LCAC-16:0 (15 μmol/L for 120 h) on HCC cells upon C646 mediated inhibition of p300 (*n* = 3). (E) Heatmap showing the genomic occupancy of H3K14ac ± 3 kb flanking TSSs in Huh7 cells. The genes shown in rows are sorted in descending order by signal strength. (F) Bioinformatics analysis filtered *KLF6* and *SMOX* as downstream targets of H3K14ac. (G) Normalized read densities for H3K14ac at the *KLF6* gene. (H) Diagram depicting the locations in the *KLF6* gene of amplicons used in chromatin immunoprecipitation (ChIP) and ChIP-qPCR assays. (I) ChIP-qPCR analysis showing *KLF6* gene occupancy by H3K14ac in HCC cells treated with 7.5 μmol/L LCAC-16:0 for 24 h (*n* = 3). Data are presented as mean ± SEM. ∗*P* < 0.05, ∗∗*P* < 0.01, ∗∗∗*P* < 0.001, ∗∗∗∗*P* < 0.0001; ns, no significance. *P* values were calculated based on one-way ANOVA.

Given that mitochondrial acetyl-CoA is an essential substrate for histone acetylation^34^, we further analyzed the effect of LCAC-16:0 on histone H3 acetylation (H3ac) levels. We found that LCAC-16:0 induced a significant increase of H3ac in HCC cells (Fig. 4A). Specifically, LCAC-16:0 increased acetylation of H3 Lys14 (H3K14ac), while not affecting H3K9ac, H3K27ac and H3K56ac (Fig. 4B and Supporting Information Fig. S4A). Increased H3K14ac was also observed in tumor tissues of DEN/CCl_4_ mice treated with LCAC-16:0 (Fig. S4B). Importantly, the increase of H3K14ac was found to be abrogated by VLCAD knockdown (Fig. 4C and Fig. S4C). To further understand the mechanism, we used C646, a histone acetyltransferase p300 inhibitor^35^^,^^36^. C646 treatment abrogated the inhibitory effect of LCAC-16:0 on HCC cells (Fig. 4D), indicating that LCACs act by modulating H3 histone acetylation.

To systematically identify candidate target genes of H3K14ac, we treated Huh7 cells with LCAC-16:0 and conducted RNA-seq and CUT&Tag assay using H3K14ac antibody. Analysis of the genome-wide distribution of H3K14ac revealed enrichment in promoter regions of specific genes (Fig. 4E). Subsequently, we identified *KLF6* and *SMOX*, which were upregulated by both LCAC-16:0 and incubation with baseline plasma of non-HCC patients (Fig. 4F, Fig. S4D and S4E). Moreover, these genes displayed elevated H3K14ac levels at their promoter regions (Fig. 4F and G, Fig. S4F). Further GSEA of RNA-seq data showed that the most suppressed gene sets by LCAC-16:0 were E2F targets, G2M checkpoint, and MYC target V1 gene sets (Fig. S4G). Given the coordinated roles of E2F, G2M, and MYC signaling, coupled with the involvement of *KLF6* in cell cycle regulation^37^^,^^38^, we focused our attention on *KLF6*. The expression of *KLF6* was confirmed by RT-qPCR (Fig. S4H and S4I). To validate H3K14ac binding in the *KLF6* promoter region, a ChIP assay was performed. ChIP assay demonstrated that the specific sites −0.5, +0.5, and +1 kb away from the transcriptional start site (TSS) of the *KLF6* gene displayed significant binding to H3K14ac (Fig. S4J). ChIP-qPCR assay further demonstrated that H3K14ac levels at specific sites of the *KLF6* promoter region were significantly enhanced after LCAC-16:0 treatment (Fig. 4H and I, Fig. S4K). Additionally, after variable splicing, *KLF6* generates three mature transcripts, wild-type, SV1, and SV2, each playing a different role in oncogenicity^39^^,^^40^. We also verified that LCAC-16:0 promoted the translation of *KLF6* but had no significant effect on its splicing process (Fig. 4H). Collectively, LCACs exert an anti-proliferation effect by promoting H3K14ac in the *KLF6* promoter region.

### 3.6 LCACs suppress HCC through KLF6/p21 pathway

**Figure 5:**
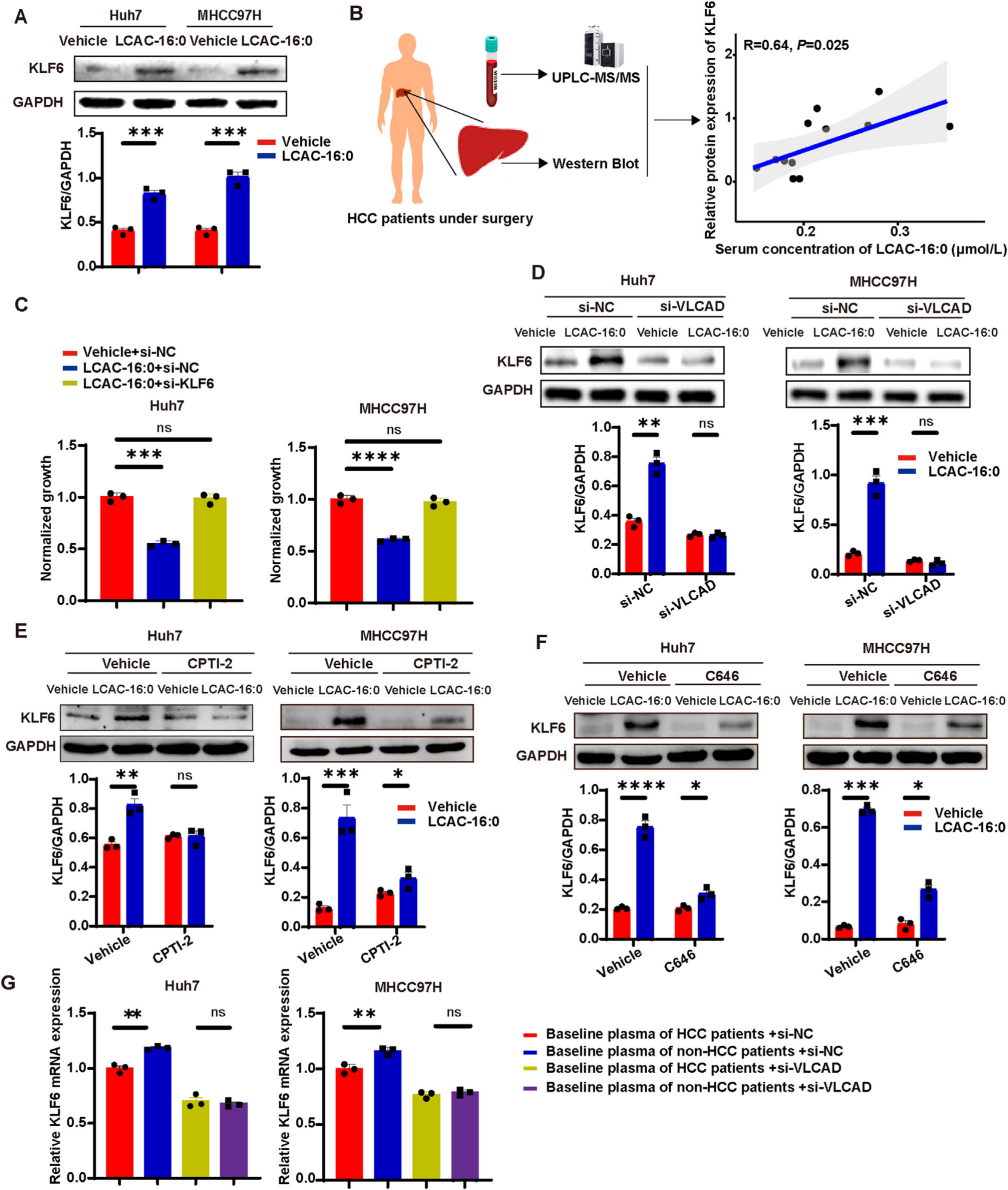
LCACs inhibit hepatocarcinogenesis *via* KLF6. (A) Western blot showing KLF6 expression levels of HCC cells treated with 7.5 μmol/L LCAC-16:0 for 96 h. (B) The correlation between relative protein expression of KLF6 in HCC tissues and plasma LCAC-16:0 levels of HCC patients (*n* = 12). (C) Inhibitory effect of LCAC-16:0 (15 μmol/L for 120 h) on HCC cells under conditions of siRNA-mediated KLF6 inhibition (*n* = 3). (D) Western blot showing KLF6 expression levels in HCC cells with si-NC or si-VLCAD in the presence or absence of LCAC-16:0 (15 μmol/L for 96 h) treatment. (E) Western blot showing KLF6 expression levels in HCC cells with or without CPTI-2 in the presence or absence of LCAC-16:0 (15 μmol/L for 96 h) treatment. (F) Western blot showing KLF6 expression levels in HCC cells with or without C646 in the presence or absence of LCAC-16:0 (15 μmol/L for 96 h) treatment. (G) qPCR showing *KLF6* expression levels in HCC cells induced by baseline plasma of HCC/non-HCC patients (48 h) with si-NC or si-VLCAD (*n* = 3). Data are presented as mean ± SEM. ∗*P* < 0.05, ∗∗*P* < 0.01, ∗∗∗*P* < 0.001, ∗∗∗∗*P* < 0.0001; ns, no significance. *P* values were calculated based on one-way ANOVA.

We further explored whether LCACs exert their anti-tumor effect through KLF6. Indeed, LCAC-16:0, LCAC-14:0, LCAC-18:0, LCAC-18:1 and LCAC-18:2 upregulated KLF6 protein expression in HCC cells (Fig. 5A and Supporting Information Fig. S5A–S5D). Consistently, in tumor tissues of DEN/CCl_4_ mice, treatment with LCAC-16:0 enhanced KLF6 expression as well (Fig. S5E). In addition, in 12 HCC patients who underwent surgery, the serum levels of LCAC-16:0 were significantly correlated with relative protein expression of KLF6 in the cancer tissues (Fig. 5B and Fig. S5F), providing further support for the conclusion that LCACs activate the KLF6 *in vivo*. To establish the direct involvement of KLF6 in the anti-tumor effect of LCACs, we knocked down KLF6 (Fig. S5G) in HCC cells and found that inhibitory effect of LCAC-16:0 on the growth of HCC cells was abolished upon KLF6 knockdown, signifying that LCACs act through KLF6 (Fig. 5C). We also verified that knockdown of VLCAD, inhibition of acetyl-CoA out of mitochondria (using CPTI-2) or inhibition of histone acetyltransferase p300 (using C646) attenuated upregulation of KLF6 by LCAC-16:0 (Fig. 5D–F). Finally, we verified whether LCACs in human plasma are responsible for the regulation of *KLF6* expression. Remarkably, the knockdown of VLCAD attenuated the difference in *KLF6* expression induced by baseline plasma of HCC/non-HCC patients (Fig. 5G).

**Figure 6:**
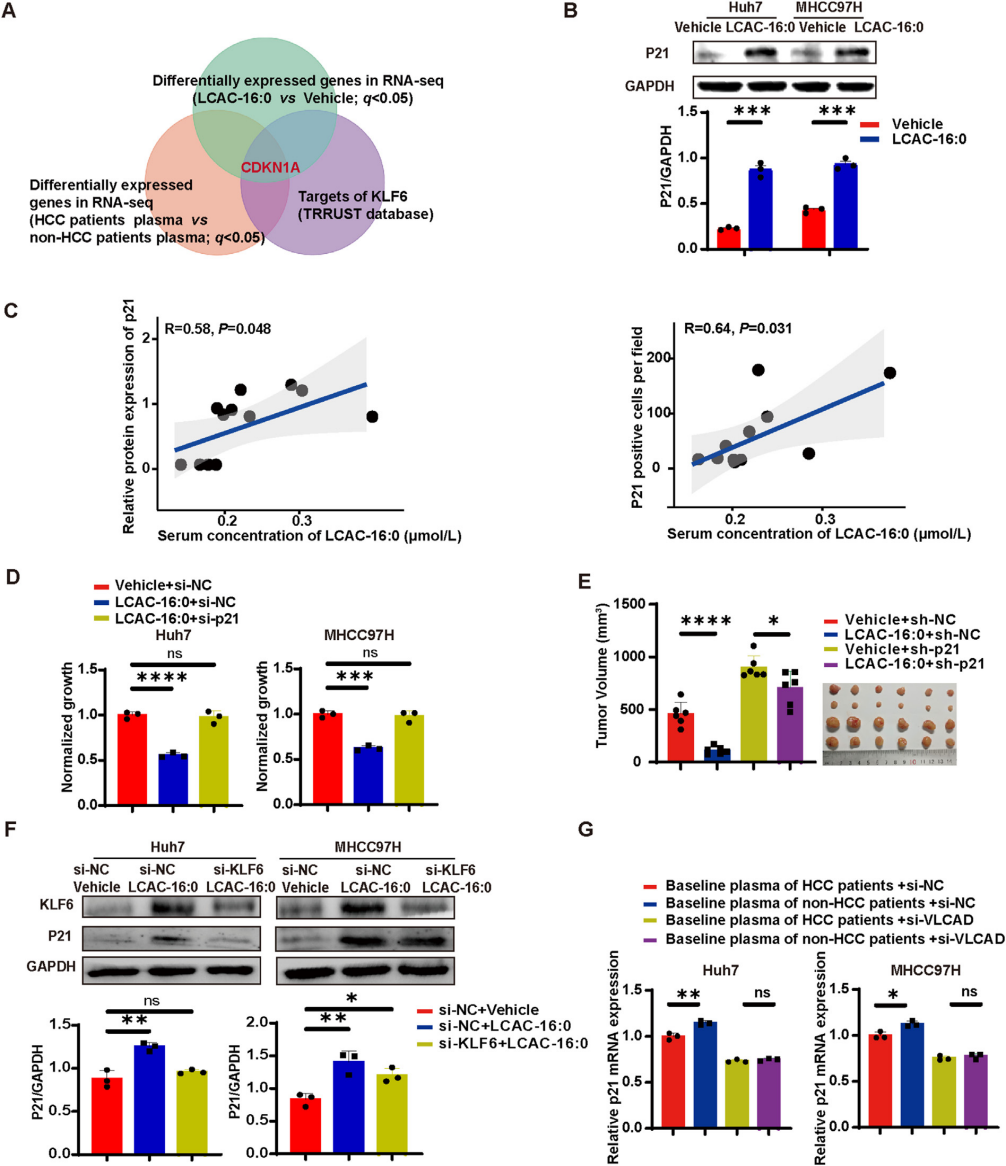
LCACs inhibit hepatocarcinogenesis *via* p21. (A) Analysis of RNA-seq data using the TRRUST database. (B) Western blot showing p21 expression in HCC cells treated with 7.5 LCAC-16:0 for 96 h. (C) The correlation between relative protein expression of p21 in HCC tissues and plasma LCAC-16:0 levels of HCC patients (*n* = 12). (D) Inhibitory effect of LCAC-16:0 on HCC cells upon siRNA-mediated p21 inhibition (*n* = 3). (E) Inhibitory effect of LCAC-16:0 (25 mg/kg/day) on MHCC97H subcutaneous tumor growth in nude mice in response to shRNA-mediated inhibition of p21 expression (*n* = 6). (F) Western blot showing p21 expression levels in HCC cells with si-NC or si-KLF6 in the presence or absence of LCAC16:0 (15 μmol/L for 96 h) treatment. (G) qPCR showing p21 expression levels in HCC cells induced by baseline plasma of HCC/non-HCC patients (48 h) with si-NC or si-VLCAD (*n* = 3). Data are presented as mean ± SEM. ∗*P* < 0.05, ∗∗*P* < 0.01, ∗∗∗*P* < 0.001, ∗∗∗∗*P* < 0.0001; ns, no significance. *P* values were calculated based on one-way ANOVA.

To further identify the critical target of the LCAC–KLF6 axis, we employed the TRRUST database^41^, a reference database of human transcriptional regulatory networks. We found that *CDKN1A* (p21), a transcriptional target of KLF6, was upregulated by both LCAC-16:0 and baseline plasma of non-HCC patients (Fig. 6A, Fig. S4A and S4B, Supporting Information Fig. S6A and B). We verified that LCAC-16:0 treatment increased the expression of p21 (Fig. 6B) without affecting subcellular localizations of p21 protein (Fig. S6C and S6D). In tumor tissues of DEN/CCl_4_ mice, p21 expression was also enhanced by LCAC-16:0 treatment (Fig. S5E). Consistently, in HCC patients, serum levels of LCAC-16:0 were strongly correlated with relative protein expression of p21 in the cancer tissues (Fig. 6C, Figs. S5F and S6E). To establish the direct involvement of p21 in the LCAC-induced inhibitory effect, we depleted p21 by siRNA (Fig. S6F), resulting in the abolishment of the inhibitory effect of LCAC-16:0 on the proliferation of HCC cells (Fig. 6D). 10.13039/100014337Furthermore, the *in vivo* inhibitory effect of LCAC-16:0 on tumor growth was significantly reduced upon p21 depletion, supporting the conclusion that LCACs act through p21 (Fig. 6E). We also confirmed that knockdown of KLF6 (Fig. 6F) and inhibition of histone acetyltransferase attenuated upregulation of p21 by LCAC-16:0 (Fig. S6G). As expected, the knockdown of VLCAD attenuated the difference in p21 expression induced by baseline plasma of HCC/non-HCC patients (Fig. 6G).

## 4 Discussion

The rapid growth in the discovery of active metabolites influencing cell physiology has been noteworthy^8^. However, it remains uncertain whether specific endogenous metabolites play an important role in the development of HCC in CHB patients, even when viral control is achieved. The Search-B cohort, a prospective and well-characterized cohort of CHB patients undergoing antiviral treatment^42^, served as the foundation for our investigation. Through metabolomics analyses integrated with functional studies, we identified LCACs as novel metabolites with clinical and mechanistic relevance to HCC development.

Previous case–control studies have revealed that HCC patients had higher blood levels of LCAC^16^^,^^17^. However, in the prospective Korean Cancer Prevention Study-II, Jee et al.^18^ showed that palmitoylcarnitine (LCAC-16:0) was associated with reduced HCC risk. We revealed that cirrhosis was positively correlated with plasma LCAC levels, which is consistent with previous studies^43^^,^^44^. Cirrhosis is a powerful risk factor for HCC^42^. Thus, cirrhosis can be a key confounder of the association between blood levels of LCAC and HCC risk. A shortcoming of previous case–control studies is the failure to balance major HCC risk factors, particularly cirrhosis^16^^,^^17^. In our study, after balancing potential confounders including cirrhosis *via* propensity score matching, we found that circulating LCAC levels are associated with reduced HCC risk.

LCACs are intermediate oxidative metabolites formed by the esterification of long-chain fatty acids and carnitine^45^. Traditionally, LCACs serve as diagnostic markers of fatty acid oxidation disorder^46^. While emerging evidence highlights that LCACs are bioactive and influence disparate aspects of pathophysiology, such as inflammation, insulin sensitivity, and protein kinase C signaling^47^, there remains a notable gap in comprehensive research into the role of LCACs in tumorigenicity. In this study, we demonstrated that LCACs at concentrations ranging from 3.75 to 7.5 μmol/L, including LCAC-14:0, LCAC-16:0, LCAC-16:1, LCAC-18:0, LCAC-18:1, and LCAC-18:2, inhibit proliferation of human HCC cells *via* KLF6/p21 pathway. However, it is crucial to note that a recent study reported contrasting results, indicating that 5 μmol/L LCAC-18:1 enhanced the self-renewal of mouse HCC cells through STAT3 activation, diverging from our findings^48^. The conflicting results raise a hypothesis that LCAC-18:1 might have paradoxical effects on hepatocarcinogenesis. While we showed that LCAC-16:0 inhibited hepatocarcinogenesis in several mouse models, the evidence regarding the comprehensive role of LCACs other than LCAC-16:0 *in vivo* is still lacking, necessitating further investigation.

LCACs have been hypothesized to regulate physiological processes by altering plasma membrane function or interacting with specific receptors^47^. Using the metabolic tracer ^13^C-(1,2,3,4)-LCAC-16:0, we demonstrated that HCC cells take up and metabolize LCACs into acetyl-CoA, a substrate for protein acetylation. While early studies reported that increased levels of acetyl-CoA and histone acetylation may be required to sustain the accelerated proliferation of cancer cells^49^, ^50^, ^51^, our study revealed contrasting results, indicating that increased levels of acetyl-CoA and H3 acetylation were associated with proliferation suppression. This divergence may be attributed to the difference in the carbon source for histone acetylation^52^. Previous studies are focused on acetyl-CoA derived from glucose and acetate^50^^,^^51^, while acetyl-CoA is also derived from LCACs, in our study. A recent study highlighted that lipid-derived acetyl-CoA can be a major carbon source for histone acetylation, and lipid-derived histone acetylation activates a specific gene expression program distinct from that induced by glucose-derived histone acetylation^52^. While further studies are needed to elucidate the detailed mechanisms of specific histone acetylation in response to different nutrients, our findings underscore that LCACs increase levels of acetylated histones and induce a specific gene expression program to suppress liver tumorigenesis.

Functional inactivation of tumor suppressor genes is a well-recognized mechanism capable of driving carcinogenesis^53^. *KLF6* is a ubiquitously expressed zinc finger transcription factor and tumor suppressor gene that suppresses growth and facilitates differentiation by p53-independent up-regulation of p21^54^, ^55^, ^56^. Given that reduction of KLF6 expression is an early and common event in hepatocarcinogenesis, it is considered a promising target for HCC prevention in patients with chronic liver disease^57^^,^^58^. However, the factors influencing KLF6/p21 expression remain to be elucidated. In this study, we demonstrated that circulating LCACs play a role in the regulation of KLF6/p21 expression, providing critical implications for HCC prevention.

The main source of circulating LCACs is still obscure. As body carnitine mainly exists in skeletal muscle, the body's acylcarnitines are speculated to be synthesized within myocytes. However, recent studies suggested that there was no correlation between the profile of acylcarnitine in plasma and skeletal muscle^59^. A previous study has revealed that LCAC-16:0 produced by blood cells was associated with the concentration of circulating acylcarnitine in healthy overweight women^60^. Similarly, we found that plasma LCAC-16:0 levels correlated well with mRNA levels of genes involved in LCAC metabolism (*Cpt1b*, *Cact*, *Bbox1*) in blood but not heart, kidney, and liver cells of mice (Supporting Information Fig. S7), suggesting blood cells might be a predominant regulator of circulating levels of LCAC.

## 5 Conclusions

In summary, this study demonstrates that LCACs serve as a protective factor against the occurrence and progression of HCC. As natural metabolites in the human body, LCACs hold significant potential for clinical application in the prevention of HCC.

## Author contributions

Kaifeng Wang: Writing – original draft, Visualization, Software, Investigation, Funding acquisition, Formal analysis, Data curation, Conceptualization. Zhixian Lan: Validation, Methodology, Investigation. Heqi Zhou: Visualization, Methodology, Investigation. Rong Fan: Resources. Huiyi Chen: Investigation. Hongyan Liang: Investigation. Qiuhong You: Investigation. Xieer Liang: Resources. Ge Zeng: Investigation. Rui Deng: Investigation. Yu Lan: Investigation. Sheng Shen: Investigation. Peng Chen: Writing – review & editing. Jinlin Hou: Resources. Pengcheng Bu: Writing – review & editing, Supervision. Jian Sun: Supervision, Funding acquisition, Conceptualization.

## Conflicts of interest

The authors declare no conflicts of interest.

## Appendix A Supporting information

The following is the Supporting Information to this article:

## Footnotes

Appendix A Supporting information to this article can be found online at https://doi.org/10.1016/j.apsb.2025.01.017.
