# Supplementary material for: Long-chain acylcarnitine deficiency promotes hepatocarcinogenesis
Source: Acta Pharm Sin B. 2025 Jan 28;15(3):1383–96. doi: 10.1016/j.apsb.2025.01.017 (PMC12069247; doi:10.1016/j.apsb.2025.01.017)
Supplement: Multimedia component 1 [file mmc1.pdf]

Supporting Information for

Original article

Long-chain acylcarnitine deficiency promotes hepatocarcinogenesis

Kaifeng Wang[a,†], Zhixian Lan[a,†], Heqi Zhou[a,†], Rong Fan[a,†], Huiyi Chen[a], Hongyan Liang[a], Qiuhong You[a], Xieer Liang[a], Ge Zeng[a], Rui Deng[a], Yu Lan[a], Sheng Shen[a], Peng Chen[b], Jinlin Hou[a,*], Pengcheng Bu[c,d,e,*], Jian Sun[a,*]

[a]*State Key Laboratory of Organ Failure Research; Key Laboratory of Infectious Diseases Research in South China, Ministry of Education; Guangdong Provincial Clinical Research Center for Viral Hepatitis; Guangdong Provincial Key Laboratory of Viral Hepatitis Research; Department of Infectious Diseases, Nanfang Hospital, Southern Medical University, Guangzhou 510515, China*

[b]*Department of Pathophysiology, Guangdong Provincial Key Laboratory of Proteomics, School of Basic Medical Sciences, Southern Medical University, Guangzhou 510515, China*

[c]*Key Laboratory of Epigenetic Regulation and Intervention, Institute of Biophysics, Chinese Academy of Sciences, Beijing 100101, China*

[d]*Key Laboratory of RNA Biology, Institute of Biophysics, Chinese Academy of Sciences, Beijing 100101, China*

[e]*College of Life Sciences, University of Chinese Academy of Sciences, Beijing 100049, China*

Received 6 August 2024; received in revised form 4 January 2025; accepted 10 January 2025

*Corresponding authors.

E-mail addresses: sunjian@smu.edu.cn (Jian Sun), bupc@ibp.ac.cn (Pengcheng Bu), jlhousmu@163.com (Jinlin Hou).

†These authors made equal contributions to this work.

1. Supporting methods

1.1. Cell lines and cultures

MIHA, L02, Huh7, Hep3B, MHCC97H, and SMMC-7721 cell lines were obtained from the Shanghai Cell Bank of the Chinese Academy of Science (Shanghai, China). HepG2 cells were obtained from the American Type Culture Collection. L02 cells were cultured in RPMI 1640 with 2%–5% FBS and the other cell lines in high-glucose DMEM with 2%–5% FBS. All cell lines were maintained at 37 °C and 5% CO_2.

1.2. Experimental animals

Mice used for experiments were age-matched. Male BALB/c nude mice and C57BL/6J mice were

purchased from Hunan SJA Laboratory Animal Co. (Changsha, China) and raised in pathogen-free facilities at the Animal Center of Nanfang Hospital under a 12 h:12 h light:dark cycle at 21–23 °C. All experimental protocols were approved by the Animal Ethics Committee of Nanfang Hospital (Ethical Committee Approval Code: NFYY-2020-1151).

1.3. Cell proliferation assay

A total of 1000 cells were seeded into 96-well plates. After 24 h, LCACs were added to set up multiple replicates for each concentration (0, 3.75, 7.5, 15 and 30 μmol/L). After incubation for 120 h, CCK-8 detection was performed.

1.4. Colony formation assay

Cells were inoculated on 6-well plates (500 cells/well) and cultured for 10 to 14 days until the formation of visible colonies. Clones were washed with PBS, fixed with 4% paraformaldehyde (Beyotime Biotechnology, Shanghai, China), and stained with 0.4% crystal violet (Beyotime Biotechnology, Shanghai, China).

1.5. Immunohistochemistry

Tumor tissues were fixed with 4% paraformaldehyde (Beyotime Biotechnology, Shanghai, China), embedded in paraffin, sectioned, and stained with hematoxylin and eosin (H&E). For immunohistochemistry, the sections were incubated overnight with primary antibodies against Ki-67 and p21.

1.6. Liver and kidney function tests of mice

Serum levels of ALT, AST, creatine, and urea were determined using 100 μL serum samples on a Catalyst Dx Chemistry Analyzer (IDEXX, Westbrook, USA) using dry-slide technology (IDEXX, Westbrook, USA).

1.7. Histone extraction and total histone H3 acetylation detection

A histone extraction kit (Invent Biotechnologies, Plymouth, USA) was utilized as follows: 0.5–5 million cultured cells were collected *via* centrifugation (600 × *g* for 5 min). Cell pellets were resuspended in 1 mL cold PBS and centrifuged at 700 × *g* for 2 min. After the complete removal of the supernatant, cell pellets were resuspended in 0.5 mL cytosolic lysate and incubated on ice for 5 min. Next, cells were centrifuged at 14,000 × *g* for 2 min and the supernatant was completely removed. Nuclear lysate (200 μL) was added to the tube, poured into prechilled filter cartridges, and centrifuged

at 16,000 × *g* for 30 s. The flowthrough in the collection tube contained extracted histone. The EpiQuik Total Histone H3 Acetylation Detection Fast Kit (Epigentek, Farmingdale, USA) was employed for total histone H3 acetylation detection according to the manufacturer's protocol.

1.8. RNA-seq analysis

Total RNA was extracted from 1×10^6 Huh7 cells using Trizol (Invitrogen, Carlsbad, USA). Total RNA was qualified and quantified using a NanoDrop and Agilent 2100 bioanalyzer (Thermo Fisher Scientific, Waltham, USA). RNA-seq was performed using an MGISEQ-2000 (The Beijing Genomics Institute, Beijing, China). Differential expression analysis was performed using DESeq2 with a Q value <0.05. Gene Set Enrichment Analysis (GSEA) was employed to examine signal pathway enrichment in different sample groups.

1.9. RNA isolation, cDNA synthesis, and quantitative real-time PCR (qRT-PCR)

Total RNA extraction was extracted using EZ-press RNA Purification Kit (EZBioscience, Roseville, USA) and cDNA was generated using PrimeScript™ RT Master Mix (Takala, Tokyo, Japan). Next, qRT-PCR was performed with SYBR Green Master Mix (Takala, Tokyo, Japan). Samples were normalized to the average level of glyceraldehyde-3-phosphate dehydrogenase (GAPDH). The relative expression of mRNA levels was calculated using the $2^{-\Delta\Delta Ct}$ method. The primer sequences are presented in Supporting Information Table S2.

1.10. Protein extraction and Western blot

Cells were lysed with proteolytic solution RIPA (mixed with protease inhibitor 1:100) (Yeasen, Shanghai, China) and incubated in a 4 °C shaker for 30 min. The soluble fractions were isolated *via* centrifugation at 4 °C (13,201 ×*g* for 30 min). For Western blot analysis, a 10-μg protein sample was subjected to 10% SDS-PAGE. After electrophoresis, the protein was transferred to PVDF membrane (Merck, Burlington, VT, USA), blocked with 5% milk for 1 h at room temperature, and incubated with primary antibodies overnight, followed by incubation with the secondary antibody for 1 h at room temperature. To detect their expression, proteins were visualized using an Enhanced Chemiluminescence (ECL) system (Bio-Rad, Hercules, DE, USA).

1.11. Immunofluorescence

Cells were fixed with 4% paraformaldehyde (Beyotime Biotechnology, Shanghai, China), treated with 0.5% Triton X-100 (Beyotime Biotechnology, Shanghai, China) at room temperature for 20 min, and blocked with 10% goat serum (Beyotime Biotechnology, Shanghai, China) at room temperature for 30

min, followed by incubation with primary antibodies against p21 (ABclonal, Wuhan, China) overnight at 4 °C. Then, cells were incubated with a secondary antibody (CST, Boston, MA, USA) at normal temperature for 1 h. Finally, slides were stained with DAPI (Yeasen, Shanghai, China) and treated with a sealing agent (Yeasen, Shanghai, China), covered with the cell climbing slices, and observed under a fluorescence microscope (Olympus, Tokyo, Japan).

1.12. Nucleoplasmic protein separation

Cells were lysed with cytoplasmic lysate (Invent Biotechnologies, Plymouth, USA). On the ice for 5 min, transferred to 1.5 mL centrifuge tubes, and mixed for 15 s, followed by centrifugation at 4 °C and 16,000 ×*g* for 5 min. Supernatant fractions (cytoplasmic components) were transferred to new centrifuge tubes. The precipitate was dissolved with 150 μL nuclear lysate (Invent Biotechnologies, Plymouth, USA), mixed for 15 s, and incubated on ice for 1 min. This step was repeated a further four times. The nuclear extract was transferred to centrifuge tubes (Invent Biotechnologies, Plymouth, USA) and subjected to centrifugation at 16,000 ×*g* for 30 s.

1.13. RNA interference

Knockdowns of VLCAD, KLF6, and p21 were achieved with siRNA technology. The VLCAD siRNA sequence was 5′-GGAUGGUUAUGCUGCAGUATT-3′, the KLF6 siRNA sequence was 5′-GCUGGACUCGGACACACUATT-3′, the p21 siRNA sequence was 5′-GAUGGAACUUCGACUUUGUTT-3′ and the negative control sense sequence was 5′-UUCUCCGAAGGUGUCACGUTT-3′. Cell transfection was performed with Lipofectamine™ 3000 (Invitrogen, Carlsbad, USA).

1.14. Construction and transfection of lentiviral vectors

Human p21 siRNA (5′-GAUGGAACUUCGACUUUGUTT-3′) was introduced into lentiviral vectors chemically synthesized using hU6-MCS-CBh-gcGFP-IRES-puromycin vector (GeneChem, Shanghai, China), designated Lv-p21 shRNA1. Empty vectors were used as controls. For stable transduction, HCC cells transduced with Lv-p21 shRNA were screened in a medium containing puromycin (5 μg/mL) (Solarbio, Beijing, China). Stable transduction was confirmed via western blot.

1.15. Data availability

The RNA-seq and CUT&Tag datasets reported in this study have been deposited in the NCBI Gene Expression Omnibus (GEO) under accession number GSE253206, GSE253207, GSE253208 in the Super Series GSE253209.

2. Supporting figures

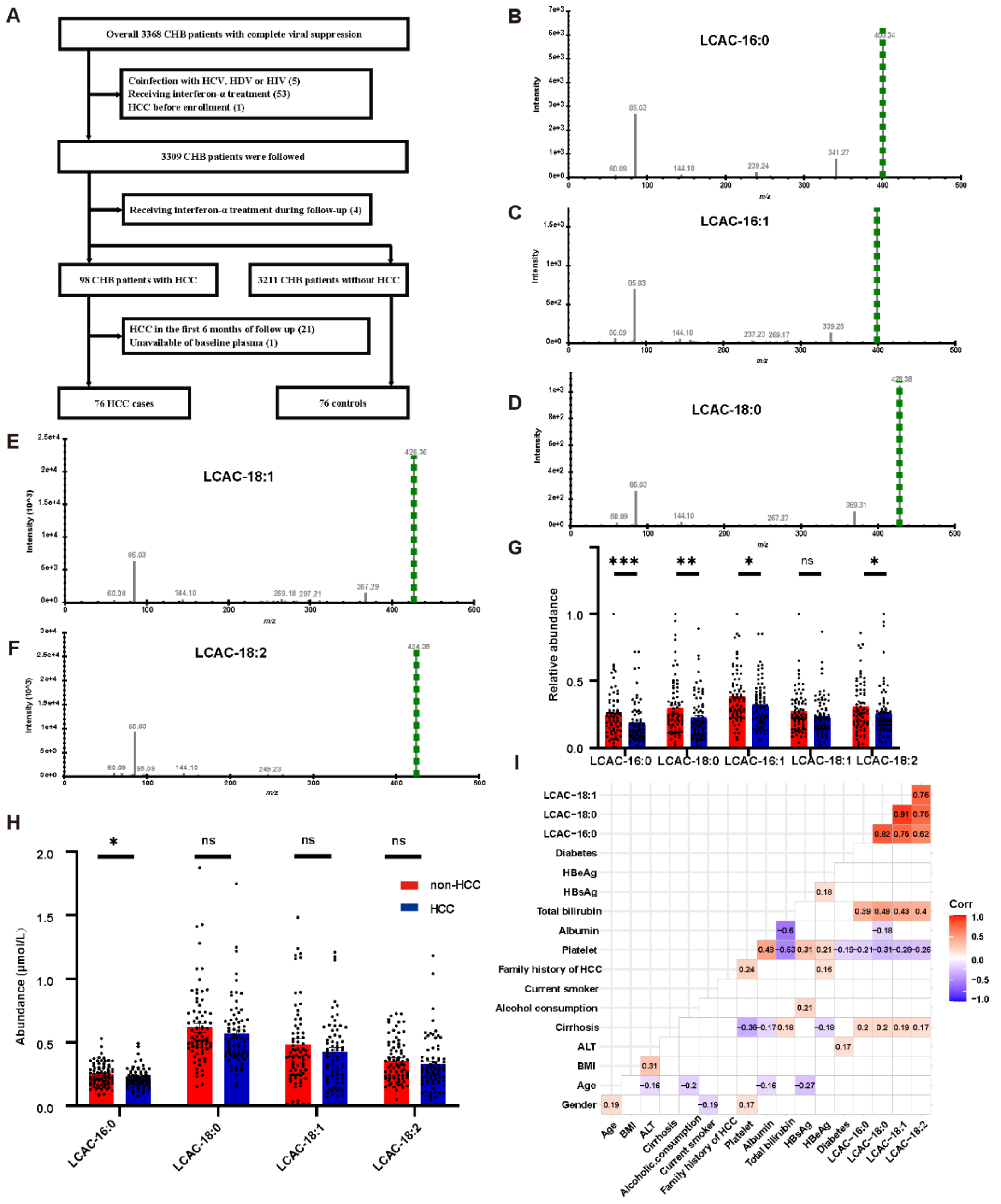

Figure S1 LCACs are negatively correlated with HCC risk.

(A) Flow chart of patients selected for analysis. **(B, C, D, E, F)** Mass spectra of LCACs in plasma. **(G)** Relative plasma levels of LCAC at baseline in CHB patients with and without HCC development during follow-up (non-HCC, *n*=76; HCC, *n*=76). **(H)** Plasma levels of LCAC at baseline in CHB patients with and without HCC development during follow-up (non-HCC, *n*=76; HCC, *n*=76). **(I)** Correlations between plasma LCAC levels and clinical parameters. Data are presented as mean ± SEM. **P*<0.05, ***P*<0.01, ****P*<0.001; ns, no significance. *P* values were calculated based on one-way ANOVA or Wilcoxon's rank-sum test.

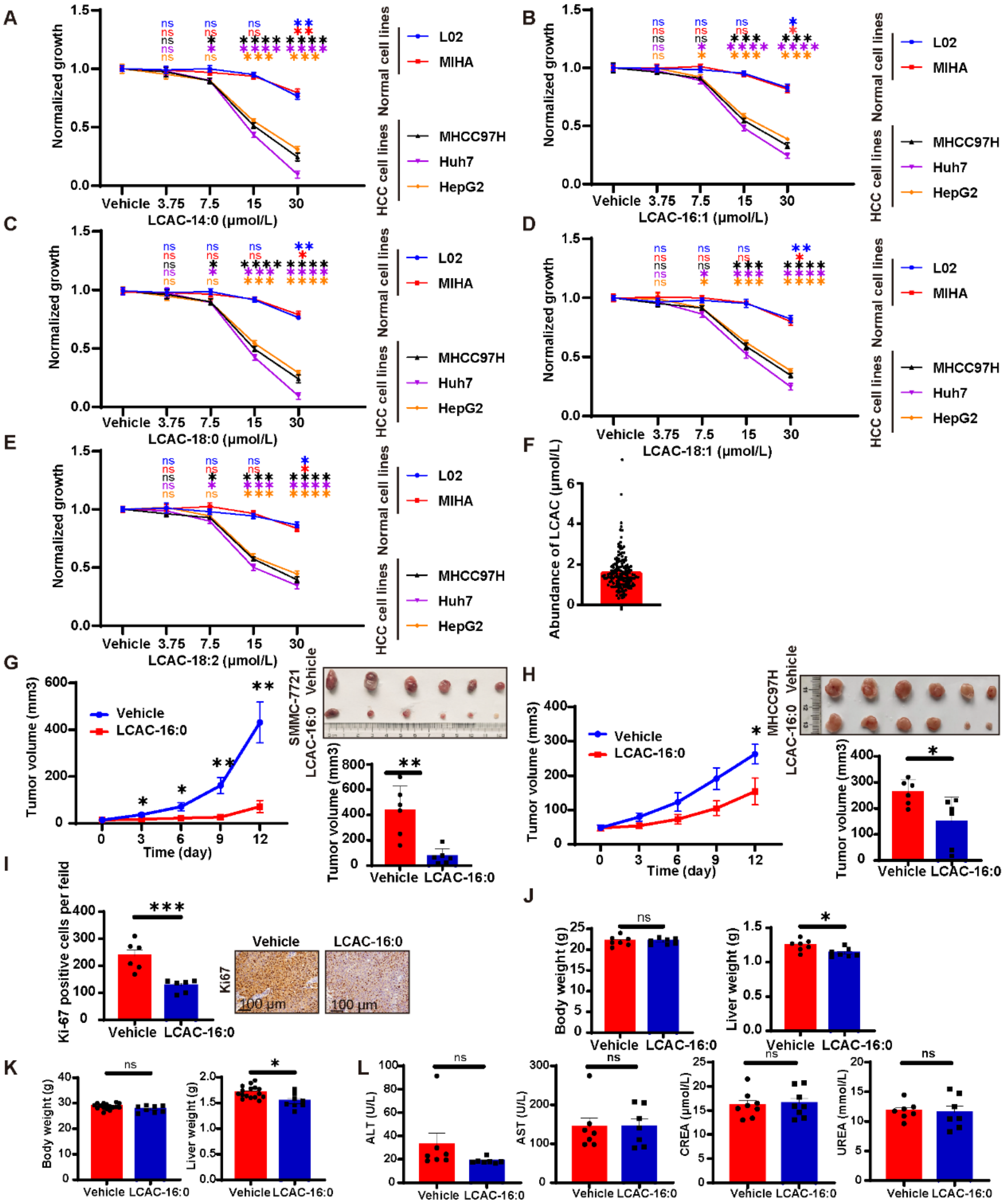

Figure S2 LCACs inhibit hepatocarcinogenesis.

(A, B, C, D, E) Dose curve of a panel of cell lines treated LCAC-14:0, LCAC-16:1, LCAC-18:0, LCAC-18:1, and LCAC-18:2 for 120 h (*n*=3). All tested doses were compared to the vehicle group. **(F)** Absolute concentration of LCAC (the sum of the concentration of LCAC-16:0, LCAC-18:0, LCAC-18:1, and LCAC-18:2) in baseline plasma of 152 CHB patients **(G, H)** Representative bright-field images and quantification of tumor volume in nude mice inoculated with MHCC97H and SMMC-7721 cells (*n*=6 for Vehicle, *n*=6 for LCAC-16:0). **(I)** Expression of Ki67 in tumor samples from nude mice inoculated with MHCC97H cells (*n*=6 for Vehicle, *n*=6 for LCAC-16:0). **(J)** Body and liver weight in AKT/Ras mice (*n*=7 for Vehicle, *n*=7 for LCAC-16:0). **(K)** Body and liver weight in

DEN/CCl4 mice (*n*=16 for Vehicle, *n*=8 for LCAC-16:0). **(L)** Serum ALT, AST, creatinine, and urea levels in LCAC-16:0/Vehicle treated mice (*n*=7 for Vehicle, *n*=7 for LCAC-16:0). Data are presented as mean ± SEM. **P*<0.05, ***P* <0.01, ****P* <0.001, *****P* <0.0001; ns, no significance. *P* values were calculated based on one-way ANOVA.

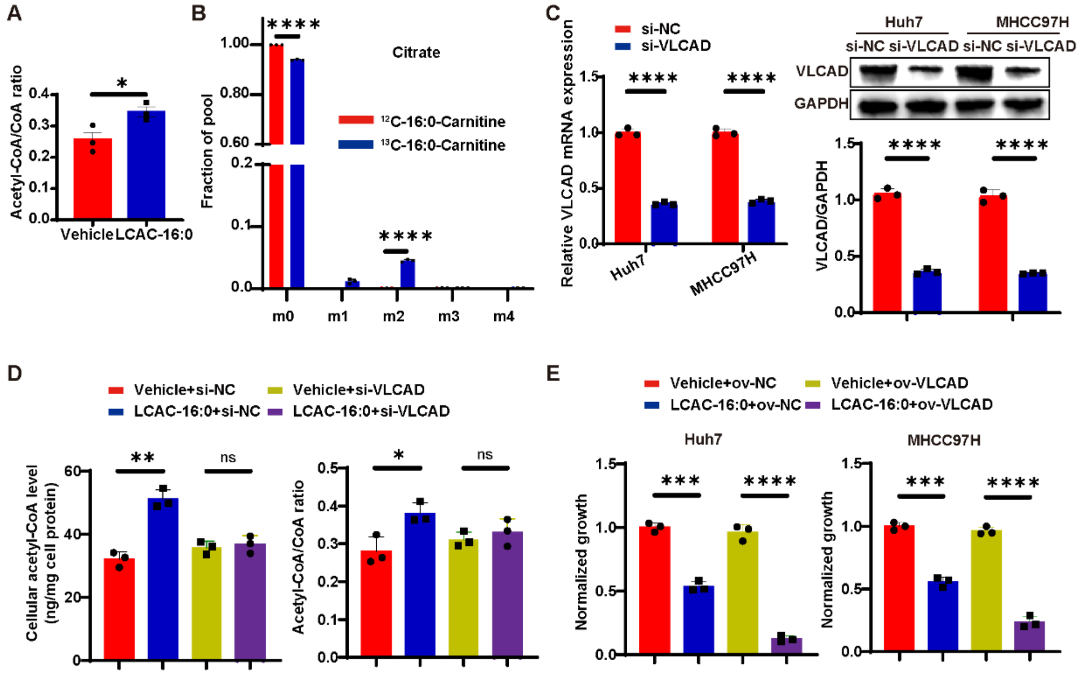

Figure S3 Uptake and metabolism of LCACs are required for their anti-tumor effect.

(A) Intracellular acetyl-CoA/CoA ratio in Huh7 cells after incubation with 7.5 μmol/L LCAC-16:0 for 24 h (*n*=3). **(B)** Isotopic tracing analysis of ^{13}C-(1,2,3,4)-LCAC-16:0 in Huh7 cells (*n*=3). **(C)** Knockdown efficiency of si-VLCAD. **(D)** Intracellular acetyl-CoA levels and acetyl-CoA/CoA ratio in Huh7 cells after incubation with LCAC-16:0 (7.5 μmol/L for 24 h) with si-NC or si-VLCAD (*n*=3). **(E)** Inhibitory effect of LCAC-16:0 (15 μmol/L for 120 h) on HCC cells upon overexpression of VLCAD (*n*=3). Data are presented as mean ± SEM. **P*<0.05, ***P*<0.01, ****P*<0.001, *****P*<0.0001; ns, no significance. *P* values were calculated based on one-way ANOVA.

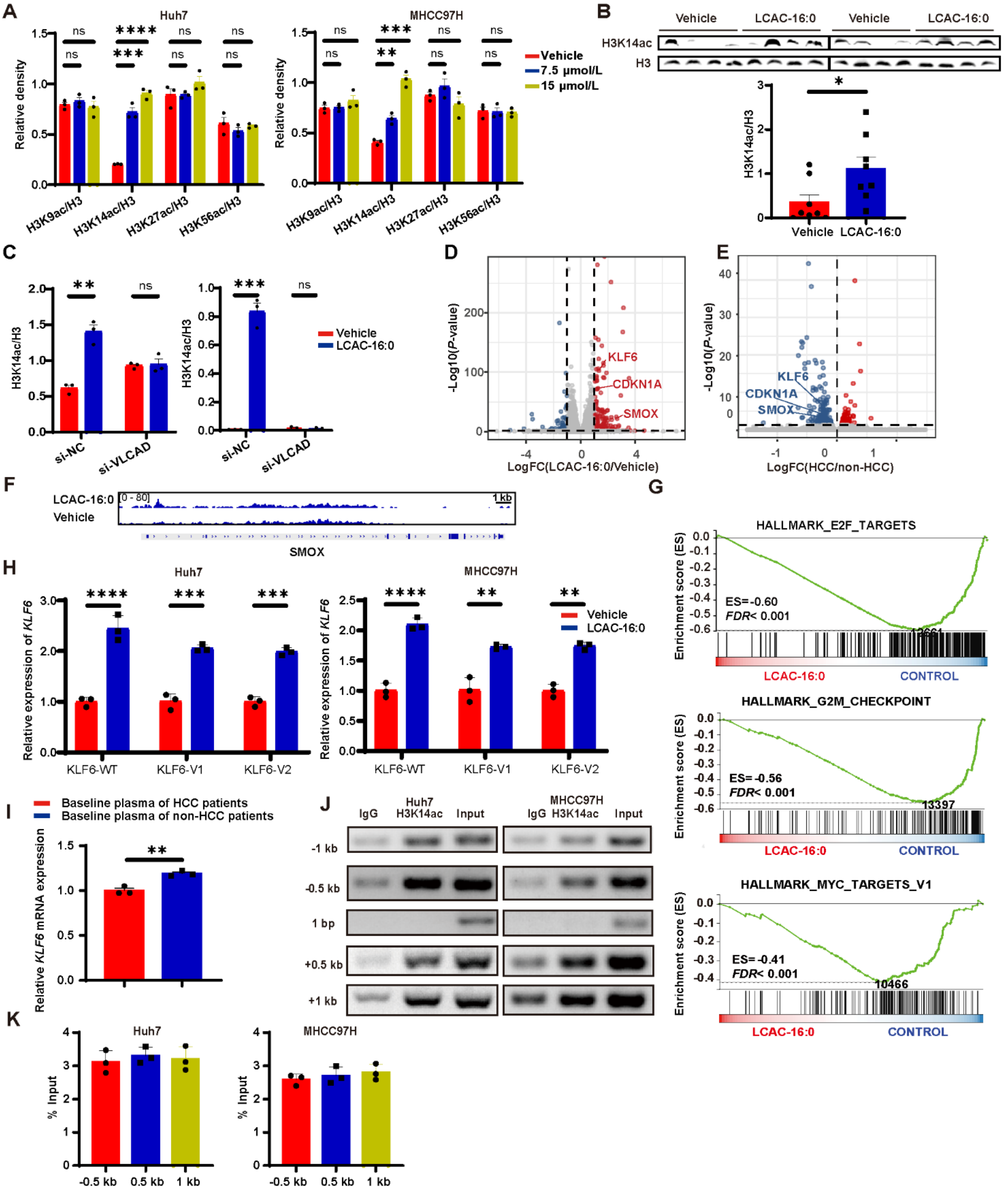

Figure S4 LCACs act through modulating H3 histone acetylation.

(A) Quantification of acetylation levels of H3K9, H3K14, H3K27, and H3K56 in HCC cells treated with indicated concentration of LCAC-16:0 for 24 h. **(B)** Western blot showing H3K14ac levels in HCC tissues of DEN/CCl_4 mice (*n*=8 for Vehicle, *n*=8 for LCAC-16:0). **(C)** Quantification of acetylation levels of H3K14 levels in HCC cells with si-NC or si-VLCAD in the presence or absence

of LCAC-16:0 (15 μmol/L for 24 h) treatment. (**D**) Volcano plot of differentially expressed genes in Huh7 cells induced by LCAC-16:0/Vehicle (*n*=3). **(E)** Volcano plot of differentially expressed genes in Huh7 cells induced by baseline plasma of HCC/non-HCC patients (*n*=3). **(F)** Normalized read densities for H3K14ac at the SMOX gene. **(G)** The top 3 signaling pathways suppressed by LCAC-16:0 in Huh7 cells. **(H)** Relative *KLF6* mRNA expression in HCC cells incubated with 7.5 μmol/L LCAC-16:0 for 48 h (*n*=3). **(I)** Relative *KLF6* mRNA expression in Huh7 cells incubated with baseline plasma of HCC/non-HCC patients for 48 h (*n*=3). **(J)** ChIP assay showing that only locations ~–0.5 kb, +0.5 kb, and +1 kb away from TSS of *KLF6* gene displayed significant binding to H3K14ac in HCC cells. **(K)** ChIP-qPCR analysis of RNA Pol II enrichment at the *KLF6* promoter region (positive control). Data are presented as mean ± SEM. **P*<0.05, ***P*<0.01, ****P*<0.001, *****P*<0.0001; ns, no significance. *P* values were calculated based on one-way ANOVA.

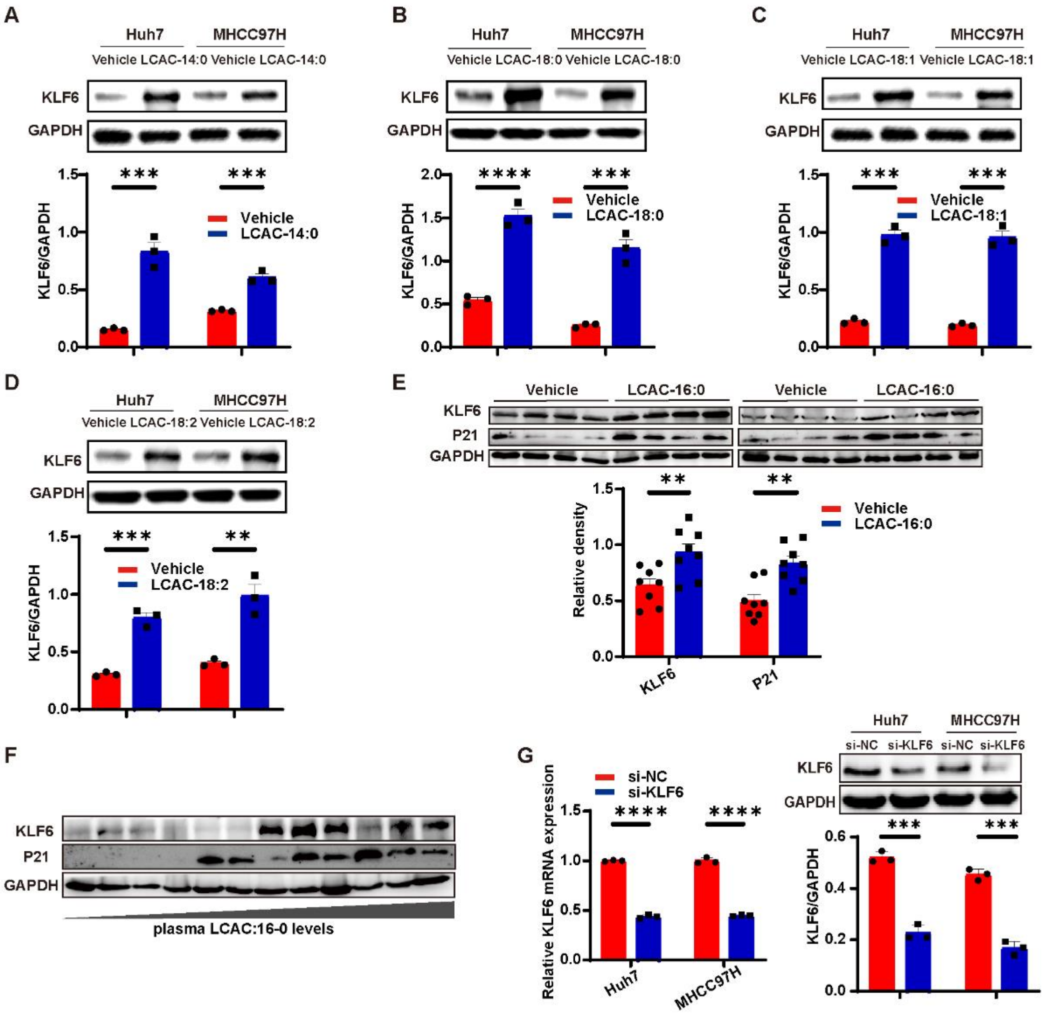

Figure S5 LCACs inhibit hepatocarcinogenesis *via* KLF6.

(A, B, C, D) Western blot showing KLF6 expression in HCC cells incubated with 7.5 µmol/L LCAC-14:0, LCAC18:0, LCAC-18:1, and LCAC-18:2 for 96 h, respectively. **(E)** Western blot showing KLF6 and p21 expression in HCC tissues of DEN/CCl_4 mice (*n*=8 for Vehicle, *n*=8 for LCAC-16:0). **(F)** Western blot showing KLF6 and p21 expression in HCC tissues of 12 HCC patients (*n*=12). **(G)** Knockdown efficiency of si-KLF6. Data are presented as mean ± SEM. ***P*<0.01, ****P*<0.001, *****P*<0.0001. *P* values were calculated based on one-way ANOVA.

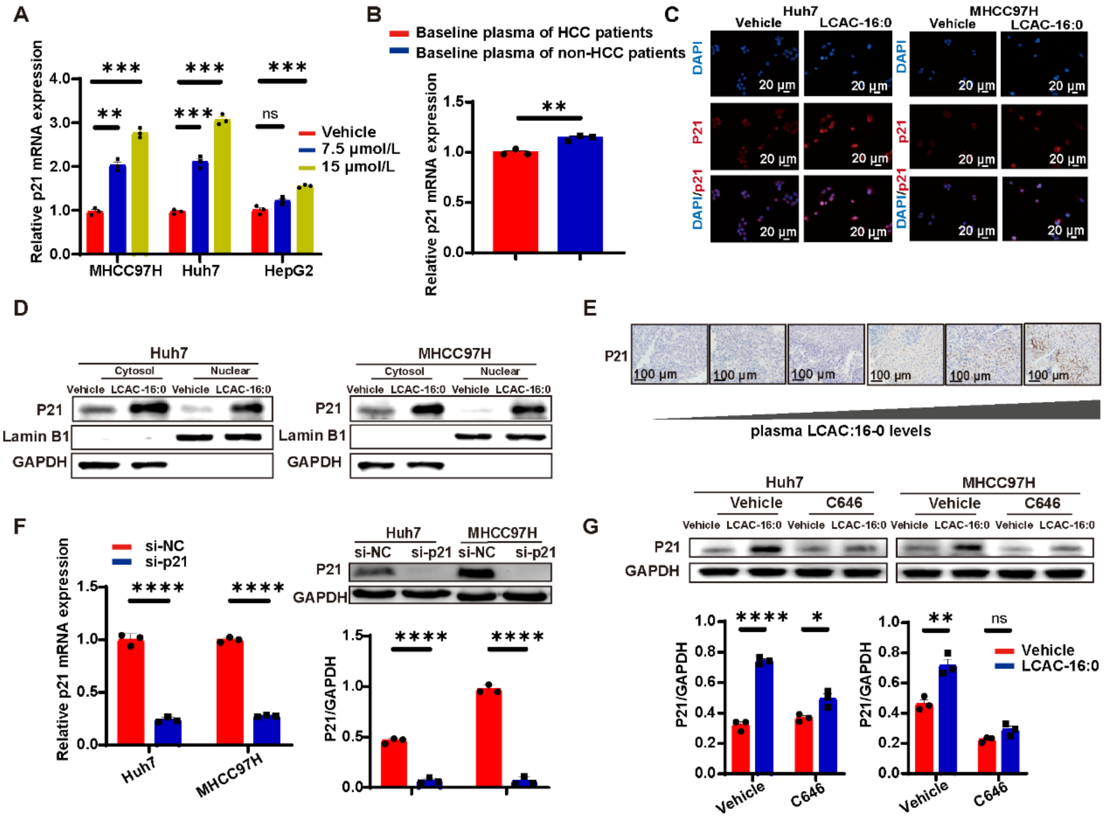

Figure S6 LCACs inhibit hepatocarcinogenesis *via* p21.

(A) Relative *p21* mRNA expression in HCC cells incubated with LCAC-16:0 for 48 h (*n*=3). **(B)** Relative *p21* mRNA expression in Huh7 cells incubated with baseline plasma of HCC/non-HCC patients for 48 h (*n*=3). **(C, D)** Protein nuclear and cytoplasmic separation experiments showing even distribution of upregulated p21 protein in the nucleus and cytoplasm following treatment with 7.5 μmol/L LCAC-16:0 for 96 h. **(E)** Immunohistochemical showing p21 expression in HCC tissues of HCC patients (*n*=12, representative shown). **(F)** Knockdown efficiency of si-p21. **(G)** Western blot showing p21 expression levels in HCC cells with or without C646 in the presence or absence of LCAC-16:0 (15 μmol/L for 96 h) treatment. Data are presented as mean ± SEM. **P*<0.05, ***P*<0.01, ****P*<0.001, *****P*<0.0001; ns, no significance. *P* values were calculated based on one-way ANOVA.

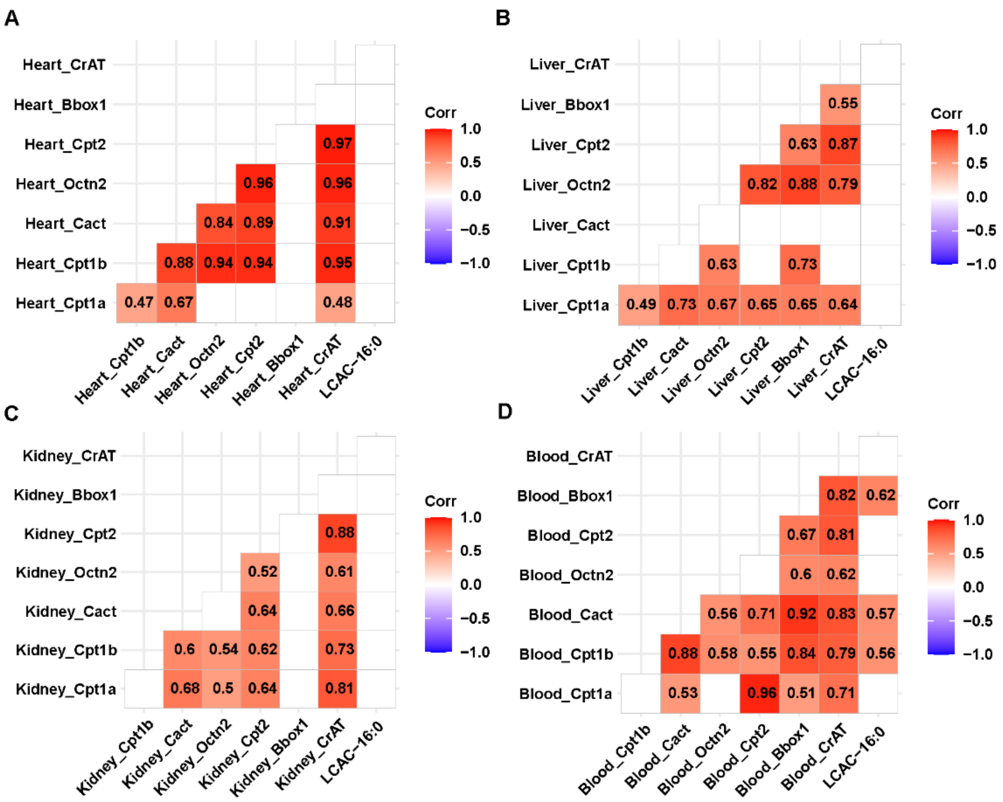

Figure S7 Correlation between plasma LCAC-16:0 levels and mRNA levels of genes involved in LCAC metabolism.

(A) Correlation between plasma LCAC-16:0 levels and mRNA levels of genes in heart cells of mice (n=14). **(B)** Correlation between plasma LCAC-16:0 levels and mRNA levels of genes in liver cells of mice (n=14). **(C)** Correlation between plasma LCAC-16:0 levels and mRNA levels of genes in kidney cells of mice (n=14). **(D)** Correlation between plasma LCAC-16:0 levels and mRNA levels of genes in the blood cells of mice (n=14).

3. Supporting tables

Table S1 Primer sequences.

I. qRT-PCR primers

Gene name	Forward	Reverse
P21	5′-AAACTAGGCGGTTGAATGAG-3′	5′-AAAGGAGAACACGGGATGAG-3′
KLF6-wt	5′-CGGACGCACACAGGAGAAAA-3′	5′-CGGTGTGCTTTCGGAAGTG-3′
KLF6-v1	5′-CCTCGCCAGGGAAGGAGAA-3′	5′-CGGTGTGCTTTCGGAAGTG-3′
KLF6-v2	5′-TCGGGGAAGCCAGGAGAA-3′	5′-CGGTGTGCTTTCGGAAGTG-3′
Cpt1a	5′-TTGGAAGTCTCCCTCCTTCA-3′	5′-GCCCATGTTGTACAGCTTCC-3′
Cpt1b	5′-GCTGCTTGCACATTTGTGTT-3′	5′-TGAGTGACTGGTGGGAAGAA-3′
Octn2	5′-AAGACCTGCAGGAAGCTGAA-3′	5′-TCCTTGTTTTTCGTGGGTGT-3′
Cact	5′-GGTGGCTGTCCAGACAAACT-3′	5′-TCCGTTTAAGAACCTCCTGG-3′
Cpt2	5′-TCTTCCTGAACTGGCTGTCA-3′	5′-GTACCCACCATGCACTACCA-3′
CrAT	5′-CTCCTGGGCTGGAGTAGATG-3′	5′-TTACAGAAGGGACTGGAGCG-3′
Bbox1	5′-TTGGCATCAATCTTGTCCTG-3′	5′-AGCTGACAAACGTGGTGAGA-3′

II. PCR primers for chromatin immunoprecipitation assay

KLF6 –10670 – –10545	5′-GCCAGAACTGCAAGAAGAAGGT-3′	5′-GAGGGGAAACTGGTAGATGG-3′
KLF6 –979 – –843	5′-TATGGAAAAAAACACACACACTAAA-3′	5′-CCTTGGAATTGGGAGAAACG-3′
KLF6 –661 – –519	5′-AAACAGAGCGGCGCAGAAGAG-3′	5′-TCCCCCCCGTCCCCTTCTAT-3′
KLF6 –112 – +66	5′-ATTGGCCGGAGCTGACATCAT-3′	5′-AACCCCTGCAAAACTTCCCT-3′
KLF6 +418 – +533	5′-GGTCGGCTGTTTGGGGTTCA-3′	5′-TCGCAGCCTGGAGGATCGAT-3′
KLF6 +894 – +1057	5′-GTCTTCCCTGCTTCTCTTGTTG-3′	5′-CCCCAAATTCCAGTTCTACG-3′

Table S2 Baseline characteristics of patients with and without HCC occurrence.

**Characteristics**	**No HCC occurrence (*n*=76)**	**HCC occurrence (*n*=76)**	***P***
**Age (year)**	49.27 (10.42)	49.88 (10.58)	0.718
**Female, *n* (%)**	7 (9.2)	7 (9.2)	1.000
**ALT (U/L)**	24.50 (18.25-38.00)	32.00 (24.00-42.50)	**0.012**
**Elevated ALT, *n* (%)**	28 (36.8)	45 (59.2)	**0.006**
**Waist circumference (cm)**	85.27 (9.65)	84.71 (8.77）	0.706
**Diabetes, *n* (%)**	8 (10.5)	11 (14.5)	0.462
**Hypertension, *n* (%)**	19 (25.0)	21 (27.6)	0.713
**HBsAg (Log_{10} IU/mL)**	2.64 (0.72)	2.55 (0.58)	0.405
**HBeAg positive, *n* (%)**	13 (17.1)	8 (10.5)	0.240
**Platelet (10^9/L)**	123.0(79.25-187.0)	131.00 (65.0-164.0)	0.240
**Total bilirubin (μmol/L)**	15.65 (11.45-19.75)	13.15 (9.25-22.02)	0.170
**Albumin (g/L)**	43.75 (40.00-46.68)	42.85 (38.00-45.43)	0.114
**Liver cirrhosis, *n* (%)**	61 (80.3)	61 (80.3)	1.000
**First-line NAs, *n* (%)**	64 (84.2)	60 (78.9)	0.403
**Current smoker, *n* (%)**	19 (25.0)	22 (28.9)	0.584
**Family history of HCC, *n* (%)**	9 (11.8)	12 (15.8)	0.481
**Alcoholic consumption, *n* (%)**	2 (2.6)	1 (1.3)	0.560
**Time of antiviral treatment* (months)**	15.66 (9.24-40.68)	26.93 (8.60-59.14)	0.182
**Time of follow-up (month)**	47.37 (37.15-56.36)	26.92 (13.27-37.27)	**<0.001**

ALT, alanine aminotransferase; NAs, nucleos(t)ide analogue; HBsAg, hepatitis B surface antigen; HBeAg, hepatitis B e antigen.

ALT, platelet, albumin, total bilirubin, time of follow-up, and duration of antiviral treatment are expressed as median (interquartile range) values and other continuous variables as mean (standard deviation).

*Time of antiviral treatment before enrollment (month).
